# Synthesis of Binary Transition Metal Nitrides, Carbides and Borides from the Elements in the Laser-Heated Diamond Anvil Cell and Their Structure-Property Relations

**DOI:** 10.3390/ma4101648

**Published:** 2011-09-28

**Authors:** Alexandra Friedrich, Björn Winkler, Erick A. Juarez-Arellano, Lkhamsuren Bayarjargal

**Affiliations:** 1 Institut für Geowissenschaften, Abt. Kristallographie, Goethe Universität, Altenhöferallee 1, D-60438 Frankfurt am Main, Germany; E-Mails: b.winkler@kristall.uni-frankfurt.de (B.W.); bayarjargal@kristall.uni-frankfurt.de (L.B.); 2 Universidad del Papaloapan, Circuito Central 200, Parque Industrial, 68301 Tuxtepec, Oaxaca, Mexico; E-Mail: eajuarez@unpa.edu.mx

**Keywords:** carbide, nitride, boride, transition metal, laser heating, diamond anvil cell, synthesis, extreme conditions, high pressure, high temperature

## Abstract

Transition metal nitrides, carbides and borides have a high potential for industrial applications as they not only have a high melting point but are generally harder and less compressible than the pure metals. Here we summarize recent advances in the synthesis of binary transition metal nitrides, carbides and borides focusing on the reaction of the elements at extreme conditions generated within the laser-heated diamond anvil cell. The current knowledge of their structures and high-pressure properties like high-(p,T) stability, compressibility and hardness is described as obtained from experiments.

## 1. Introduction

Transition metal carbides, nitrides and borides are a large and complex group of industrially relevant compounds with outstanding physical properties. The combination of metals with light covalent-bond forming atoms like B, C and N often leads to materials which not only have a high melting point, but also have a very low compressibility and high hardness compared with the pure metal. Examples for binary transition metal carbides, nitrides and borides with remarkable properties are the extremely high melting points of HfC (4201 K [1]) and TaC (4223 K [1]), the large bulk modulus of OsB (453 GPa [2]), and the ultra-hardness of ReB${}_{2}$ (H${}_{v}$ = 30–48 GPa [2,3,4]), WB${}_{4}$ (H${}_{v}$ = 46 GPa [2]) and MoN (H${}_{v}$ = 38.5 GPa [5]). Structurally unusual features are observed for the ultra-incompressible nitrides of Os, Ir and Pt, which contain dinitrogen units within their crystal structures. TaC has a comparatively high transition temperature into a superconducting state with T${}_{c}$ = 10.3 K [1]. Hence, it is of interest to understand their structure-property relations. The structure-property relations of those nitrides and carbides of groups IV–VI and of the transition metal borides, which can by synthesized at ambient pressure, have been studied extensively, both experimentally and theoretically [1,2,6,7,8].

**Figure 1:**
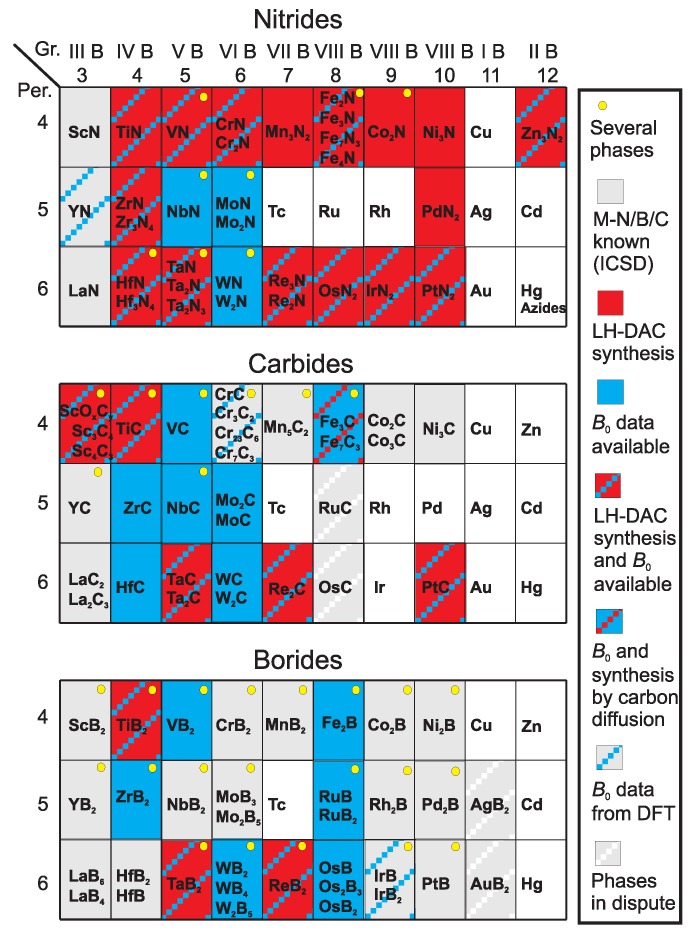
Overview of known binary transition metal nitrides, carbides, and borides discussed here.

Figure 1 gives an overview over most of the known binary transition metal nitrides, carbides, and borides. This review will focus on the more recent work related to the synthesis of novel compounds in this group of materials from the elements in laser-heated diamond anvil cells. This research area has attracted attention since the synthesis of cubic *γ*-Si${}_{3}$N${}_{4}$ in 1999 [9], and as is obvious from Figure 1, most work was directed to the synthesis of transition metal nitrides, with fewer studies of transition metal carbides and borides.

The study of reactions is, however, not only important for the synthesis of new materials, but it is of more general use to understand processes occurring in laser-heated diamond anvil cells at extremely high pressures and temperatures. For example, conflicting melting curves have been reported from melting studies of metals at extreme (p,T)-conditions. It now seems that during laser heating there is a significant diffusion of carbon from the diamonds into the reaction chamber. The subsequent contamination of the metal has a drastic influence on its melting temperature and this may be the origin of the large discrepancies in the experimentally determined melting curves of some metals and alloys (e.g., [10,11]).

This review is structured as follows: first, a brief summary of the experimental approach of interest here, namely the laser-heated diamond anvil cell, will be provided. Increasingly, experimental studies are complemented by theoretical studies, and extensive efforts are underway to predict novel compounds. Hence, the most commonly used modelling approach, based on density functional theory, is introduced. We will then summarize studies on transition metal nitrides, followed by a chapter on transition metal carbides and a chapter on transition metal borides. In each section, we will first discuss the binary compounds of period 4 transition metals, followed by those of period 5 and period 6 transition metals. The isostructural zirconium and hafnium nitrides are discussed within one section, while palladium nitride is discussed together with other platinum group nitrides of period 6 which contain dinitrogen units within their crystal structures. In a concluding part, we attempt to correlate structure-property relations across the three groups of compounds by considering the unit cell volume, density, bulk modulus and hardness of a compound.

## 2. Experimental Approach

### 2.1. General Considerations

This review is restricted to investigations of the formation of transition metal nitrides, carbides and borides in the laser-heated diamond anvil cell (DAC) at high pressure and temperature, and alternative methods, such as the use of large volume presses, will not be discussed. However, it is obvious that while the (p,T)-range attainable with toroidal or multi-anvil cells is significantly smaller than the range available in the DAC, the advantage of being able to study samples half a million times larger will be crucial for some experiments.

The experiments reviewed here commence from mixtures of the elements. Alternatives, such as using precursors, may be required to obtain meta-stable structures, but this approach is not established for the synthesis of the compounds of interest here. While fine grained graphite and boron are readily available and an obvious choice as a starting material, the metals are available as foils or powders. The foils often have the advantage that they already have an appropriate thickness. Also, some fine grained metal powders are highly flammable while foils often can be handled outside a glove box. From a practical point of view, compact mixtures of graphite and metal can be more easily produced from powders. However, a homogeneous mixture of the light elements like carbon or boron with the heavy transition metals of period 6 of the elements is difficult to obtain due to the significant difference in weight. There is clearly a need for new sample preparation techniques to be developed, such as depositing a thick carbon film on a metal foil. Experiments involving nitrogen are technically relatively straightforward as compressed nitrogen can be loaded in an autoclave at elevated pressure of 1–3 kbar, or as liquid nitrogen below the sublimation temperature of nitrogen. These are established techniques, as nitrogen is often used as a pressure-transmitting medium. As nitrogen serves as both a reactant and the pressure-transmitting medium there is no contact problem with the transition metal during the high-(p,T) reaction.

### 2.2. Laser-Heated Diamond Anvil Cell Experiments

Laser heating of mixtures of graphite or boron and metal is comparatively easy, as both materials absorb radiation over a very wide range of energies. Hence, in contrast to experiments with transparent samples, there is no need to use lasers emitting long wavelength radiation, such as CO${}_{2}$ lasers, but instead the more common near-infrared lasers such as Nd:YAG, Nd:YLF or fiber lasers can be employed. The sample must be thermally insulated from the diamonds. This can be achieved by either placing a compacted sample on a few grains of material or coating the diamonds, e.g., with NaCl or KCl, which can then serve as a pressure medium as well in case of carbide or boride formation, or using single-crystalline sapphire chips. It should be noted here that the use of hygroscopic salts requires careful preparation as even trace amounts of water may influence the result of the experiment. Due to the strong absorption of the laser radiation by the opaque samples—most transition metals absorb well except for highly reflecting metals such as gold—only moderate laser power (<20 W) is required to achieve bright hot spots. Double-sided laser heating is now acknowledged as a prerequisite to minimize thermal gradients. Synthesis experiments of carbides at pressure and temperature conditions within the stability field of diamond might result in the transformation of graphite into polycrystalline diamond, which does not absorb near-infrared laser radiation. When nitrogen is used as the pressure-transmitting medium, only the metal is absorbing near-infrared laser radiation and serves as the heat source for inducing reactions with nitrogen.

*In situ* observations of the reactions require the use of an appropriate experimental facility at a synchrotron source. A number of experimental stations, where double sided laser heating can be performed, are available at, e.g., the ALS (Berkeley, beamline 12.2.2 [12]), the ESRF (Grenoble, beamline ID27 [13]), the APS (Chicago, GSECARS, beamline 13-ID-D [14]), SPring-8 (Nishiharima, beamline BL10XU [15]), and PETRA III (Hamburg, beamline P02.2 [16]). Typically, either image plates or CCDs have been employed as detectors, but increasingly flat panel detectors are used. In the near future, it is likely that new detector systems (so called “pixel detectors” such as the PILATUS detector) will become available, which will enable faster data collection, will have a larger dynamic range and higher spatial resolution, and will allow an energy discrimination. While such detectors are already used for diffraction studies using comparatively long wavelengths, their efficiency for high energy X-rays, which are required to overcome absorption by the diamonds in DACs and cover a significant part of reciprocal space, is currently still in need for improvement.

Depending on the beamline, pressures are determined with either on-line or off-line spectrometers using the ruby fluorescence method [17,18] and/or from the known equation of state of the pressure-transmitting medium, such as NaCl, Ar or nitrogen, or of the metal used as the sample. Pressures determined independently by ruby fluorescence and an internal standard will typically agree to within 1–2 GPa. Usually laser-heating experiments start at pressures not much lower than 10 GPa in order to avoid graphitization of the diamond anvils of the pressure cell during laser heating. In comparison to the pressure determination, the temperature determination is experimentally more challenging. At high temperatures, the current method of choice is the measurement of the emitted thermal radiation and fit of its spectral distribution using Planck’s or Wien’s law or the two-color pyrometry. Details of this procedure are described in Benedetti and Loubeyre [19]. This method has, however, significant short-comings, which are discussed by Benedetti and Loubeyre [19], as the fundamental physical processes of the emission process are ill-constrained. As the samples deviate from ideal black bodies, they have to be treated as grey bodies with temperature gradients. Axial temperature variations (in depth) are generally not considered in the temperature fits of emission spectra and may have a strong influence on the temperature determination. Further, the grey-body approximation assumes the emissivity to be independent of the wavelength. However, the consideration of the wavelength-dependence of the emissivity and its dependence on temperature are crucial for the correct extraction of temperatures from spectral fits. Complementary techniques, such as time-resolved Raman spectroscopy or life-time measurements of electronic excitations are currently developed [20], but are not widely applied yet.

### 2.3. Characterization of the Synthesis Products

Samples obtained from diamond anvil cell experiments usually are extremely small with typical dimensions of 40 × 40 × 10 *μ*m${}^{3}$. In order to increase the signal-to-noise ratio from the sample and to avoid overlapping artifacts stemming from the surrounding materials, micro-beam techniques have to be used for their characterization.

(i) The compressibility can be determined from the measurement of the pressure dependence of the unit-cell parameters using X-ray diffraction at a synchrotron source. In general this can be achieved right after the synthesis of the material while it is still pressurized within the DAC or on the recovered sample, which is again loaded in a DAC. The latter is more complex, but has the advantage that a highly hydrostatic pressure-transmitting medium like Ne or He can be chosen for the experiment.

(ii) The vibrational properties can be measured by micro-Raman or infrared spectroscopy. Depending on the composition of the isolating layer between the sample and the diamond, e.g., sapphire chips, and the type of the diamonds used for laser heating, it might be preferable to recover the sample first. Conventional micro-Raman and micro-IR spectrometers have typical spot sizes of 2–10 *μ*m and the data acquisition is rapid.

(iii) Micro X-ray fluorescence, with a spatial resolution of 1 *μ*m, is available at some synchrotron beamlines. This allows to map the distribution of elements in samples enclosed within diamond anvil cells, e.g., [21].

(iv) White-beam micro-X-ray diffraction with a spotsize of around one micron allows to scan the sample and rapidly determine the orientation of grains across the sample, e.g., [22]. As the orientation of neighbouring grains will not be correlated, this technique then allows to determine the grain size by a mapping of grain orientations. The capability of this method has already been demonstrated on a postperovskite sample loaded at Mbar pressure within the diamond anvil cell [23].

(v) Transmission electron microscopy (TEM) and electron energy loss spectroscopy (EELS) analyses of recovered samples can be used to determine the composition, oxidation states and unit cells of the products. Even structure solutions are possible on nano-sized crystals [24,25,26]. Hence, this promising method could be applied on recovered samples and would be especially useful if the material consisted of light and heavy elements. The increasing availability of focused ion beam sources for the preparation of tiny samples for such experiments also opens new possibilities [27,28].

(vi) A large variety of other micro-beam techniques, most of which are related to recent developments at 3rd generation synchrotrons (X-ray absorption spectroscopy, nuclear inelastic scattering, *etc*.), are in principle suitable to further characterize the products of reactions in diamond anvil cells, but they have not often been employed during *in situ* synthesis studies and will therefore not be discussed here further.

An interesting property which is often studied is the hardness. However, this is a property which depends both on the actual measurement technique and the sample microstructure. Hence, there have been controversial discussions about individual values (see e.g., discussion on hardness of rhenium diboride [3,29,30] and on the meaning of the word “ultra-hard” [31]).

The analysis of the chemical composition of samples recovered from laser-heating DAC experiments is a major challenge with respect to light elements due to the small sizes of the samples. While in many laser-heating DAC studies the chemical composition of the reaction products was not analysed, the importance of chemical analysis is undisputed and this point should be addressed with a higher priority in future studies. The high affinity of some metals to oxygen is a wide-spread problem during sample storage and sample preparation for the synthesis of pure binary transition metal nitrides, carbides and borides (e.g., [32,33]). This often causes the incorporation of minor amounts of oxygen in the synthesis products, which may influence the resultant properties of the materials (e.g., [34]). A similar problem has only recently been reported for the diffusion of carbon from the diamond anvils into the sample during laser-heating experiments (e.g., [10,11]). The consequence of carbon contamination and its effect on the properties of the resultant transition metal nitrides and borides has not been considered in most studies so far.

## 3. Computational Approaches

Density functional theory (DFT) is currently the method of choice for the calculation of structure-property relations of crystalline materials. An introduction to DFT calculations is given by Martin [35]. While the prediction of unknown phases in order to guide experiments is still a major challenge, a very large number of studies addressing structure-property relations of transition metal carbides, nitrides and borides have been published in the last decade. For known structures calculations of properties such as the bulk modulus are often straightforward with established packages such as ABINIT [36], QUANTUM ESPRESSO [37], CASTEP [38] or similar codes. Employing codes using plane waves as a basis set in conjunction with pseudopotentials is relatively straightforward, as the numerical convergence only depends on two parameters (*k*-space sampling and number of plane waves) and the error due to the pseudopotential can be tested by calculating known properties of a range of related structures. The inaccuracies introduced by using one of the approximations for the exchange-correlation energies (e.g., the local density approximation [39,40], the generalized gradient approximation [41] or a hybrid functional such as B3LYP [42]) are well controlled. Lattice parameters will generally differ from experimental values by not more than 1%–3% [43], elastic stiffness coefficients of incompressible structures are reproduced to a few GPa [44], computed band gaps generally have substantial error [45], while phonon frequencies are correct to within a few cm${}^{-1}$ [46,47]. The application of DFT-based methods to correlated systems, including many iron-containing compounds, often requires the use of techniques beyond “standard” DFT, such as LDA/GGA+U (e.g., [48]). The structural complexity of transition metal nitrides, carbides and borides is generally limited (see Table 1, Table 2 and Table 3), which facilitates such model calculations. However, from experiments it is well known that all properties are influenced to some extent by non-stoichiometry (e.g., the compressibility of TaC${}_{x}$, Table 2, or the hardness of thin films [49,50]). As appropriate supercell calculations quickly become computationally rather expensive, and as many experimental studies address the non-stoichiometry in a cursory manner only, discrepancies between experimental and computed values are to be expected.

## 4. Transition Metal Nitrides

The structure-property relations of the nitrides of groups IV–V transition metals have been summarized by Pierson [1]. The nitrides of groups IV–VI have very similar properties to the respective carbides, *i.e.*, they are hard and wear-resistant with high melting points and good chemical resistance. They are similar in structure and composition. However, the nitrides are not as refractory as the carbides. While nitrides of groups IV and V have melting points above 2000 K, those of group VI dissociate rapidly at high temperature (≈1200 K). Hence, they are chemically less stable. Compared to the pure metals the mononitrides of groups IV and V have higher melting points except for VN. The differences are smaller for the group V metal nitrides.

The synthesis of binary nitrides as bulk materials has been reported from experiments for all transition metals of periods 4–6 except for Cu, Tc, Ru, Rh, Ag, Cd, Au and Hg (Figure 1) [51]. Reaction experiments of the transition metals with nitrogen in the laser-heated DAC have already been performed in the majority of transition metal-nitrogen systems (Figure 1). The synthesis of transition metal nitrides in the laser-heated diamond anvil cell is straightforward when loading nitrogen as compressed gas, which fills all free volume in the pressure chamber and hence allows the best contact with the metal. Further, nitrogen is present in excess, which allows the reaction of the entire metal and results in the formation of a nitrogen-saturated phase under these conditions. The high-pressure chemistry of nitride-based materials has been reviewed in Horvath-Bordon *et al.* [52]. Here, we will focus on reactions of the elements in the laser-heated diamond anvil cell and summarize the most recent results.

### 4.1. Period 4 (3*d*) Transition Metal Nitrides

**Figure 2:**
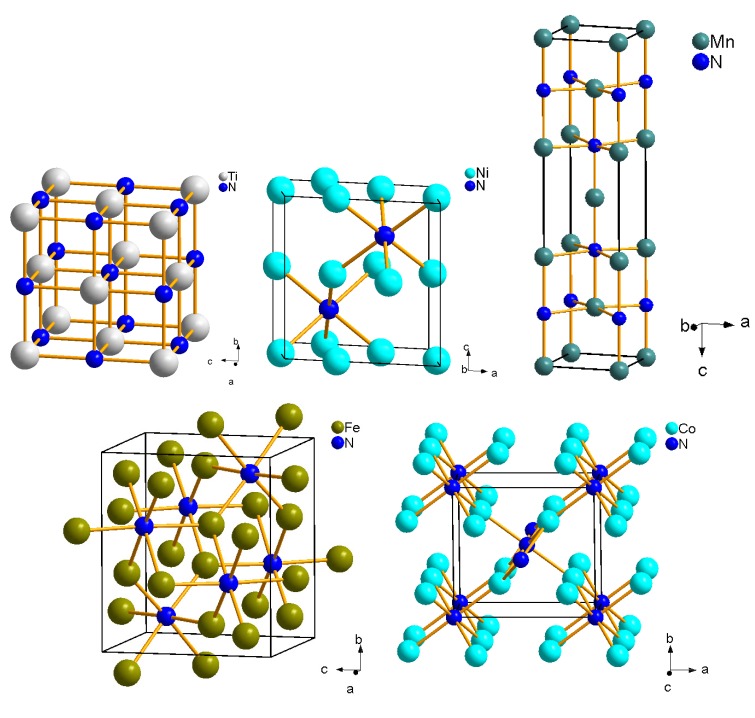
From top left to bottom right: Crystal structures of TiN (NaCl-type structure, $Fm\bar{3}m$), Ni${}_{3}$N (*P*6${}_{3}$22), Mn${}_{3}$N${}_{2}$ (*I*4/mmm), Fe${}_{2}$N (Pbcn) and Co${}_{2}$N (Pnnm) [53].

**Figure 3:**
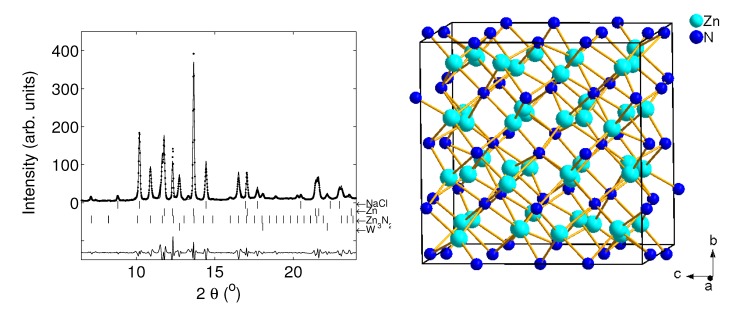
Left: Zn was loaded with nitrogen as reactant and pressure-transmitting medium and with NaCl for insulation in a DAC and compressed up to 10 and 24 GPa. After subsequent laser heating Zn${}_{3}$N${}_{2}$ was synthesized. The diffraction pattern was measured after pressure release at 0.8 GPa (*λ* = 0.4958 Å). Diffraction lines of tungsten stem from the gasket. Right: Crystal structure of Zn${}_{3}$N${}_{2}$ ($Ia\bar{3}$) consisting of edge-sharing ZnN${}_{4}$ tetrahedra.

Most transition metals of period 4 of the periodic table of elements were reacted with nitrogen in a laser-heated DAC at ≈10 GPa and ≈1800 K by Hasegawa and Yagi [53]. The samples were then recovered at ambient conditions and analysed *ex situ* using powder X-ray diffraction. The direct nitriding reaction led to the formation of TiN, VN and CrN with NaCl-type structures ($Fm\bar{3}m$, *a* = 4.235(1) Å, 4.1361(7) Å and 4.135(1) Å, respectively). The formation of TiN from the elements had already been observed earlier at pressures up to 25 GPa and 3000 K, confirming the high stability of TiN with NaCl-type structure [54]. For the transition metals with higher atomic numbers the N/metal ratio decreases successively by the formation of Mn${}_{3}$N${}_{2}$ with IrU${}_{2}$C${}_{2}$-type structure (*I*4/mmm, *a* = 2.994(1) Å and *c* = 12.499(5) Å), Fe${}_{2}$N with PbO${}_{2}$-type structure (Pbcn, *a* = 4.423(4) Å, *b* = 5.531(3) Å, *c* = 4.821(3) Å), Co${}_{2}$N with CFe${}_{2}$-type structure (Pnnm, *a* = 4.662(9) Å, *b* = 4.332(5) Å, *c* = 2.749(9) Å) and Ni${}_{3}$N with O${}_{3}$Re-type structure (*P*6${}_{3}$22, *a* = 4.594(8) Å, *c* = 4.325(9) Å) (Figure 2) [53]. Copper, however, did not react with nitrogen at these conditions. We have reacted zinc with nitrogen at 10 and 24 GPa during laser heating. Zn${}_{3}$N${}_{2}$ ($Ia\bar{3}$, anti-bixbyite-type structure, *a* = 9.769 Å) was formed as the stable phase (Figure 3). There is no reason to believe that scandium nitrides, which can be obtained at ambient pressure, will not also form at high pressures, and hence the only remaining challenge is the synthesis of a copper nitride.

### 4.2. Zirconium and Hafnium Nitrides

**Figure 4:**
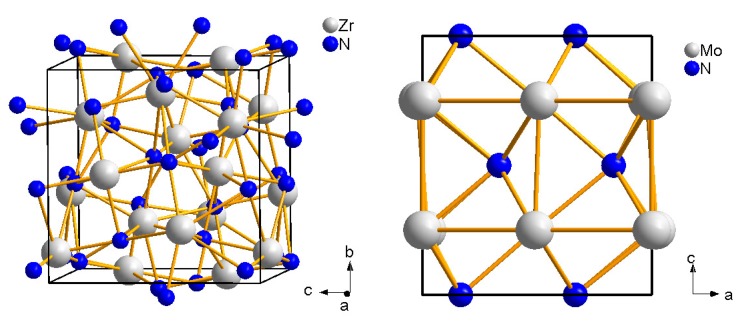
Crystal structures of c-Zr${}_{3}$N${}_{4}$ ($I\bar{4}3d$, left) consisting of edge-sharing ZrN${}_{8}$ polyhedra [54] and of *δ*-MoN ($P6_{3}mc$, right) [51].

The reaction of zirconium and hafnium with nitrogen was achieved at pressures up to 18 GPa and temperatures up to 3000 K in the laser-heated DAC and recovered samples were analysed *ex situ* by powder X-ray diffraction, TEM, energy-dispersive X-ray spectroscopy (EDX), and Raman spectroscopy [54]. c-Hf${}_{3}$N${}_{4}$ was formed at 18 GPa and 2800 K, while the formation of *c*-Zr${}_{3}$N${}_{4}$ was observed between 15.6–18 GPa and 2500–3000 K. Both phases crystallize in the cubic Th${}_{3}$P${}_{4}$ structure ($I\bar{4}3d$, *a* = 6.701(6) Å and 6.740(6) Å, respectively, Figure 4, left), where the cations are eightfold coordinated by nitrogen anions. The structural model for the new high-(p,T) phases was confirmed by Rietveld refinement and Raman spectroscopy.

### 4.3. Molybdenum Nitrides

Although molybdenum nitrides have not been synthesized by reaction of the pure elements in the laser-heated DAC so far, the reactivity of molybdenum nitrides under nitrogen atmosphere was studied in the laser-heated DAC. *γ*-Mo${}_{2}$N ($Fm\bar{3}m$, NaCl-type, *a* = 4.18 Å) was pressurized up to 54 GPa and heated at 2000 K [55]. While *γ*-Mo${}_{2}$N was stable at these conditions under argon atmosphere, additional reflections of the ordered hexagonal phase *δ*-MoN ($P6_{3}mc$, FeS(2H)-type, *a* = 5.733 Å and *c* = 5.608 Å, Figure 4, right) were observed at similar conditions under nitrogen atmosphere. Hence, Machon *et al.* [55] concluded that *δ*-MoN appears to be a limiting phase for nitrogen incorporation. No formation of new compounds with higher nitrogen content such as Mo${}_{3}$N${}_{4}$ or Mo${}_{3}$N${}_{5}$ was observed.

### 4.4. Tantalum Nitrides

Many tantalum nitrides of different stoichiometries are known and we have discussed the sometimes conflicting nomenclature in a recent study [56]. At ambient pressure, the successive nitridation of tantalum at high temperatures (<3200 K) first leads to the formation of TaN${}_{x}$ with x< 0.15 and space group $Im\bar{3}m$, where the lattice parameter is significantly expanded compared to pure tantalum (with $a_{Ta}$ = 3.311 Å) depending on the nitrogen content. Then *β*-Ta${}_{2}$N is formed, which is either described in space group $P6_{3}/mmc$ (*a* = 3.05 Å and *c* = 4.919 Å) as the disordered high-*T* phase or in $P\bar{3}$1*m* (*a* = 5.285 Å and *c* = 4.919 Å) as an ordered low-*T* phase with Fe${}_{2}$N structure. And finally nitridation results in hexagonal *ε*-TaN with CoSn-type structure in space group $P\bar{6}2m$ (*a* = 5.196 Å and *c* = 2.911 Å) [57].

**Figure 5:**
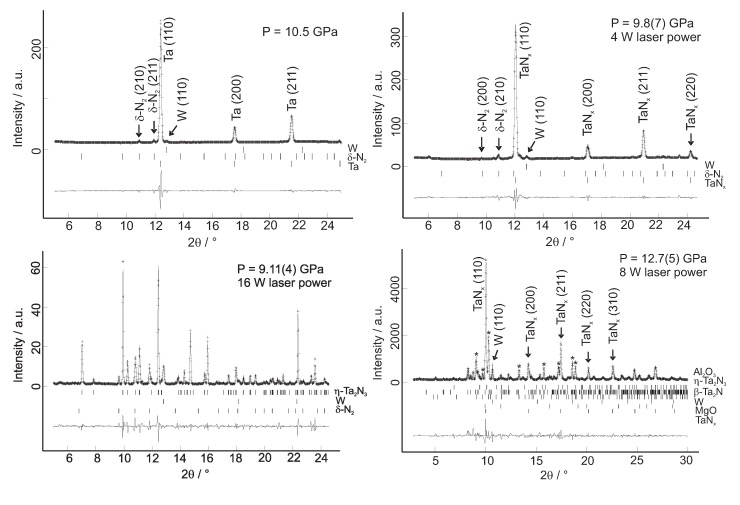
Refined powder X-ray diffraction patterns (*λ* = 0.4958 Å) for the unreacted tantalum foil at 10.5 GPa (top left), the TaN${}_{x}$ after laser alignment (top right), and the fully reacted *η*-Ta${}_{2}$N${}_{3}$ after strong laser heating at 9 GPa (bottom left) [56]. Bottom right: Refined powder X-ray diffraction pattern (*λ* = 0.4132 Å) for another reacted tantalum sample, where after laser alignment at 12.7(5) GPa the formation of TaN${}_{x}$, *β*-Ta${}_{2}$N (reflections marked by stars) and a small amount of *η*-Ta${}_{2}$N${}_{3}$ was observed [56]. Diffraction lines of tungsten stem from the gasket.

**Figure 6:**
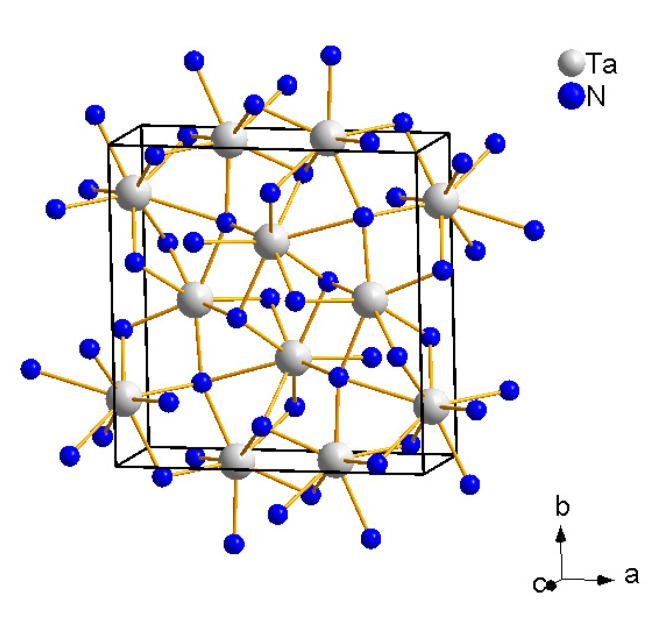
Crystal structure of *η*-Ta${}_{2}$N${}_{3}$ (Pbnm) consisting of edge-sharing TaN${}_{7}$ and TaN${}_{7+1}$ polyhedra [32].

We have shown by *in situ* experiment that the nitridation process of tantalum also occurs at high pressures (up to 27 GPa) and temperatures (>1600–2000 K) within the laser-heated DAC (Figure 5) [56]. All the tantalum reacted immediately in all steps of nitridation, pure tantalum was not present any more in either of six independently loaded and heated samples. The nitridation sequence of tantalum at about 10 GPa and high temperature commences with the formation of cubic TaN${}_{x}$ ($Im\bar{3}m$) with 0.05 < *x* < 0.10 upon brief double-sided laser heating at < 1000 K. This is deduced from a moderate, but significant, enlargement of the tantalum unit cell. Further laser heating at increasing temperature leads to the disappearance of this phase and the formation of *β*-Ta${}_{2}$N. Heating the sample further in the DAC results in orthorhombic *η*-Ta${}_{2}$N${}_{3}$ (Pbnm, U${}_{2}$S${}_{3}$-type structure, *a* = 8.191(2) Å, *b* = 8.183(2) Å, *c* = 2.9823(3) Å, Figure 6) [32,56] as the final reaction product and stable high-(p,T) phase in the presence of excess nitrogen up to at least 27 GPa and 2000 K. The exact composition of these phases was not investigated, and they may be non-stoichiometric. *η*-Ta${}_{2}$N${}_{3}$ can be recovered to ambient conditions and was also obtained by previous quench experiments using Ta${}_{3}$N${}_{5}$ as the starting material at 11 < *p* < 20 GPa and 1773–1973 K within a multi-anvil press [32]. As Ta${}_{3}$N${}_{5}$ was prepared *via* a reaction of Ta${}_{2}$O${}_{5}$ with ammonia by Zerr *et al.* [32], a small contamination of the starting product (Ta${}_{3}$N${}_{4.89}$O${}_{0.15}$) of the multi-anvil press experiments and, hence, also of the final high-(p,T) phase *η*-Ta${}_{2-2/3x}$(N${}_{1-x}$O${}_{x}$)${}_{3}$, where 0 ≤x≤ 0.05, with oxygen was unavoidable. The substitution of small amounts of oxygen for nitrogen may play an important role in stabilizing orthorhombic *η*-Ta${}_{2}$N${}_{3}$ according to Jiang *et al.* [34], who predicted the mechanical instability of pure orthorhombic *η*-Ta${}_{2}$N${}_{3}$ using DFT-based calculations. They also proposed a tetragonal Ta${}_{2}$N${}_{3}$ phase to be more stable than the orthorhombic phase at lower pressures and its transformation to *η*-Ta${}_{2}$N${}_{3}$ at 7.7 GPa. In the laser-heating study by Friedrich *et al.* [56], however, a tantalum foil was used as starting material and, hence, contamination with oxygen is expected to be less relevant and may only affect the surface of the foil.

In the laser-heated DAC experiments (up to 27 GPa, >1600–2000 K) neither the formation of Ta${}_{3}$N${}_{5}$-I nor of a novel Ta${}_{3}$N${}_{5}$-II phase was observed. A Ta${}_{3}$N${}_{5}$-II phase had been predicted to be the product of a reaction of *ε*-TaN with nitrogen at pressures above 17–25 GPa at 2800 K [58]. Also, the tetragonal Ta${}_{2}$N${}_{3}$ phase, which had been predicted to be the stable phase below 7.7 GPa by Jiang *et al.* [34] was not observed. Furthermore, neither the formation of *ε*-TaN nor of the high-pressure phase *δ*-TaN were observed. This implies that while *δ*-TaN may be obtained from hexagonal *ε*-TaN [59], which is the stable phase at ambient conditions and is obtained by the nitriding process at high temperature [57], it is not accessible from direct reaction of the elements at elevated (p,T)-conditions.

### 4.5. Rhenium Nitrides

**Figure 7:**
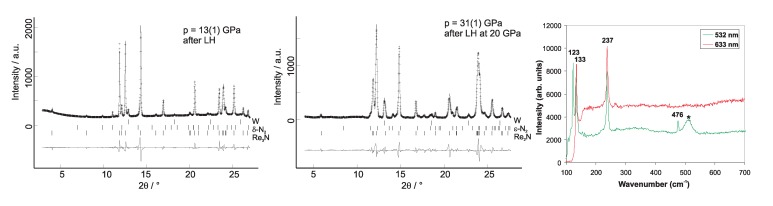
Left and center: Powder X-ray diffraction patterns (*λ* = 0.4958 Å) of Re${}_{3}$N after laser heating at 1700(200) K at 13(1) GPa (left), and of Re${}_{2}$N at 31(1) GPa after reaction during laser heating at 20(2) GPa and pressure increase (center) [60]. Diffraction lines of tungsten stem from the gasket. Right: Raman spectra of Re${}_{3}$N applying a HeNe laser (top line, 633 nm, 50 mW) and a Nd/YAG laser (bottom line, 532 nm, 20 mW) [61]. The broad band indicated by a star is attributed to fluorescence.

High pressure seems to be a prerequisite for the formation of rhenium nitrides. While no reaction of rhenium foil with nitrogen was observed at 10.0(5) GPa and temperatures up to 2800 K [60], a novel rhenium nitride, Re${}_{3}$N ($P\bar{6}m2$, *a* = 2.78(1) Å, *c* = 7.152(4) Å) is formed at 13(1) GPa and temperatures of 1700(200) K (Figure 7). Re${}_{3}$N is stable on successive laser heating up to 2250(150) K at pressures between 10.5 and 16 GPa. During laser heating at about 2000 K at pressures of 20(2) GPa another novel rhenium nitride, Re${}_{2}$N ($P6_{3}/mmc$, *a* = 2.83(5) Å, *c* = 9.88(1) Å) is formed and becomes the stable phase. Both phases can be recovered at ambient conditions [60].

**Figure 8:**
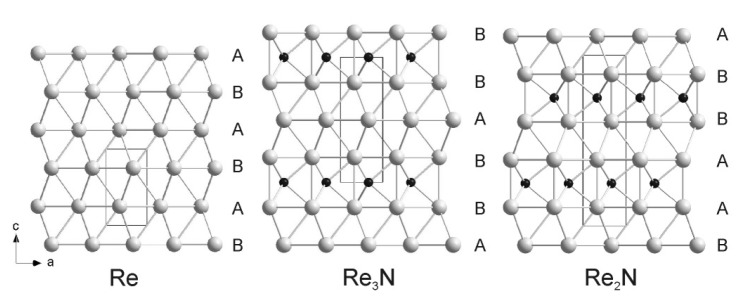
Comparison between the crystal structures of Re, Re${}_{3}$N and Re${}_{2}$N [60].

The crystal structures of Re${}_{3}$N and Re${}_{2}$N were obtained from DFT-based calculations on a sequence of trial structures and confirmed by Rietveld refinement of the rhenium positions. Re${}_{3}$N has an ABB stacking sequence of rhenium atoms, Re${}_{2}$N an AABB one, identical to that of Re${}_{2}$C [62]. Nitrogen is located between the AA or BB layers only. The crystal structures of Re, Re${}_{3}$N, and Re${}_{2}$N are shown in Figure 8. The overall agreement between observed and calculated structural parameters is so satisfactory that we conclude that the compounds are either stoichiometric or very nearly so.

**Figure 9:**
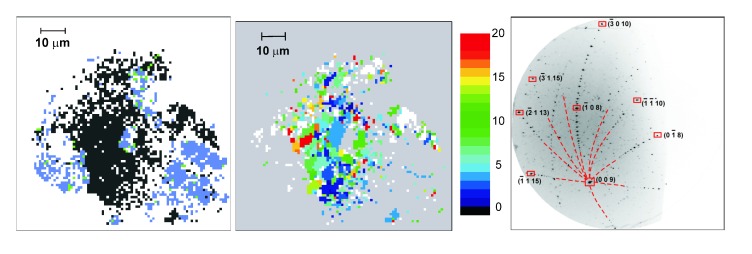
From left to right: Typical results from white beam X-ray microdiffraction showing a map of the phase distribution (Re${}_{3}$N and Re in black and blue areas, respectively), the distribution of the grains and their sizes (≈3–8 *μ*m) derived from the *c*-axis orientation of Re${}_{3}$N, and an image indexed with the unit cell of Re${}_{3}$N [60]. The indices of a few reflections are shown as examples. The red dashed curves indicate extensions of the indexed Laue zones.

X-ray microdiffraction was performed on the recovered reaction products for characterization at the ALS. From the indexation of Laue patterns of single grains the unit cells could be confirmed, which were obtained from powder X-ray diffraction and proposed for Re${}_{3}$N and Re${}_{2}$N. A white-beam X-ray mapping across the samples and subsequent automatic indexation of the images resulted in maps of the phase distribution within the samples and of the grain orientations (Figure 9), which show the polycrystalline nature of the reaction products (grain sizes between ≈3–8 *μ*m).

The proposed structural model of Re${}_{3}$N was further confirmed by micro-Raman spectroscopy on recovered samples [61]. All four Raman-active modes were observed in perfect agreement with DFT (Figure 7, right). This further confirms the presence of single nitrogen atoms within the crystal structure, which implies that nitrogen dissociates during the synthesis of the rhenium nitrides.

### 4.6. Osmium, Iridium, Platinum and Palladium Nitrides

**Figure 10:**
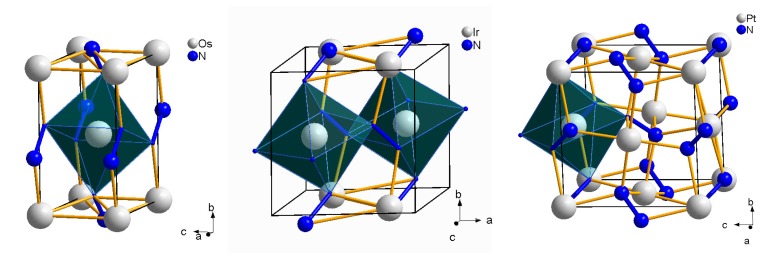
Crystal structures of orthorhombic OsN${}_{2}$ of marcasite-type structure (left, [68]), monoclinic IrN${}_{2}$ of CoSb${}_{2}$- or arsenopyrite-type structure (center, [69,70]) and cubic PtN${}_{2}$ of pyrite-type structure (right, [64]).

The formation of nitrides of Os, Ir, Pt and Pd requires much higher pressures than that of the previously discussed transition metal nitrides, *i.e*., conditions above 45 GPa and 2000 K. The formation of OsN${}_{2}$ was reported at 43 GPa and >2000 K [63], of IrN${}_{2}$ above 47 GPa and 1600 K [64] up to 64 GPa [63], of PtN${}_{2}$ at above 45–50 GPa and 2000 K [64,65], and of PdN${}_{2}$ at above 58 GPa and temperatures at about 800–900 K [66,67]. The most interesting feature of their crystal structures is that they contain nitrogen as single-bonded dinitrogen units, which is in contrast to the other known transition metal nitrides formed at extreme conditions and was observed first for PtN${}_{2}$ (Figure 10).

OsN${}_{2}$ has an orthorhombic unit cell with *a* = 2.714(2) Å, *b* = 4.910(5) Å, *c* = 4.102(3) Å and lacks Raman activity [63]. The 1:2 stoichiometry of OsN${}_{2}$ was first suggested from *ab initio* structural optimizations by tentatively testing a variety of different nitrogen starting positions within the metal lattice parameters and metal positions, which were obtained from powder X-ray diffraction [63]. Finally, a marcasite-type structure (Pnnm, Figure 10, left) was proposed for OsN${}_{2}$ from DFT calculations by Montoya *et al.* [68], giving excellent agreement between the experimental and theoretical data, e.g., unit cell parameters, bulk modulus and lack of Raman activity, which was inferred to be due to its metallicity [63,68]. Hence, the presence of dinitrogen units in the crystal structure could be confirmed.

For iridium nitride a relatively complex Raman spectrum was measured with at least 11 modes, which indicates that the structure is of lower symmetry than the structure of cubic PtN${}_{2}$ (see below) [63,66]. Scanning electron microscopy (SEM) and EDX yielded a nitrogen composition for both IrN${}_{x}$ and PtN${}_{x}$ with 0.6 <x< 2 [64]. The 1:2 stoichiometry of IrN${}_{2}$ was again suggested from *ab initio* structural optimizations in a similar way to OsN${}_{2}$ [63]. It was supported by the observation of similarities of the Raman spectrum with that of PtN${}_{2}$, particularly in the frequency region of the dinitrogen units [63]. For IrN${}_{2}$ a hexagonal lattice (*a* = 3.966(4) Å and *c* = 6.958(7) Å) was proposed first by Young *et al.* [63]. In a subsequent experimental study Crowhurst *et al.* [66] found a monoclinic unit cell for IrN${}_{2}$ and proposed the baddeleyite-type structure ($P2_{1}/c$). This structure-type has 18 Raman-active modes, the calculated ones agreeing well with the 11 measured modes. Independently, the CoSb${}_{2}$ structure ($P2_{1}/c$, *a* = 4.809 Å, *b* = 4.858 Å, *c* = 4.848 Å, *β* = 108.25 ${}^{\circ }$, Figure 10, center), which is related to the arsenopyrite structure and very close to the baddeleyite structure, but contains Sb${}_{2}$ units in contrast to the oxide, was predicted for IrN${}_{2}$ from DFT calculations of the Raman spectra and comparison with experimental data [69,70].

Cubic platinum nitride was first reported with composition PtN crystallizing in the zinc-blende type structure ($F\bar{4}3m$, *a* = 4.8041(2) Å) [65]. However, only one Raman mode is active in the zinc-blende structure, while four Raman modes in the range 790–1050 cm${}^{-1}$ were observed in the Raman spectrum of platinum nitride. A comparison of Raman spectra by Crowhurst *et al.* [64] showed close relationship to that of pyrite, FeS${}_{2}$ (Figure 10, right), which has five Raman-active modes. Hence, they proposed a composition PtN${}_{2}$ with dinitrogen units in the crystal structure, which was confirmed by SEM and EDX [64], and by DFT calculations [71].

A comparison of the measured Raman spectra of PtN${}_{2}$ and PdN${}_{2}$ indicates that palladium nitride crystallizes in the pyrite structure as well [66,67]. The formation pressure of PdN${}_{2}$ is very high, while the required temperature is much lower than for the formation of PtN${}_{2}$. In contrast to all other transition metal nitrides discussed in this section PdN${}_{2}$ cannot be recovered to ambient conditions, but decomposes at pressures below ≈13 GPa or even at higher pressures if temperature is increased [66,67].

Finally, the crystal structures of IrN${}_{2}$ and PtN${}_{2}$ are isotypic to those of IrP${}_{2}$ [72] and PtP${}_{2}$ [73], which can be synthesized at ambient pressure and high temperature. Hence, it is of interest which high-pressure phases IrP${}_{2}$ and PtP${}_{2}$ would form. The study of their high-(p,T) behaviour would provide further insight to the structural stability of these materials and could serve as analogue for the behaviour of the respective nitrides at even higher (p,T)-conditions.

### 4.7. Mechanical Properties of the Transition Metal Nitrides

The experimentally obtained values for the bulk modulus and hardness of several binary transition metal nitrides are summarized in Table 1. The group IV and period 4 transition metal nitrides have experimentally determined bulk moduli <300 GPa where the iron nitrides are the most compressible. The bulk moduli of Nb, Mo, Ta, Os, and Pt nitrides range around 350 GPa, while the highest incompressibility (*B* ≈ 400 GPa) is observed for the nitrides of the heaviest transition metals, *i.e*., of Re and Ir. The comparably low bulk modulus of W${}_{2}$N (240 GPa, Table 1) might be correlated with the high value for $B^{\prime }$ = 11.7, and hence cannot be directly compared to the other values with $B^{\prime }$ < 6. It should be noted, though, that for a NaCl-type structure $B^{\prime }$ = 11.7 is highly unusual and that this value is based on a single experimental study only [74].

The highest hardness is reported for molybdenum and tungsten nitrides. However, no experimentally determined hardness values exist for the Re, Os, Ir, Pd and Pt nitrides, which were solely synthesized in the laser-heated DAC. This is due to the small sample volume available.

Experiments have shown that the stoichiometry of nitrides is positively correlated with the nitrogen partial pressure during synthesis, for example during the sputtering process of thin films and deposition of coatings [49,50]. This implies that the equilibrium synthesis products at extreme pressures and temperatures as generated in the laser-heated DAC should be nearly stoichiometric compounds but this requires additional experimental verification.

**Table 1:** Properties of selected transition metal nitrides. $B_{0}$ is the bulk modulus and $B^{\prime }$ its pressure derivative. ${}^{*}$ indicate phases obtained at high-(p,T) only.

	Compound	Space group	Structure	$B_{0}$ (GPa)	$B^{\prime }$	Ref.	Hardness (GPa)	Ref.
Group III	ScN	$Fm\bar{3}m$	NaCl					
	YN	$Fm\bar{3}m$	NaCl	DFT: 157	3.5	[75]			
	LaN	$Fm\bar{3}m$	NaCl					
Group IV	TiN	$Fm\bar{3}m$	NaCl	277–289		[76,77,78]	18–21	[1,76,79]
	*δ*-ZrN	$Fm\bar{3}m$	NaCl	248		[80]	15.8–17.4	[1,80]
	c-Zr${}_{3}$N${}_{4}$${}^{*}$	$I\bar{4}3d$	c-Th${}_{3}$P${}_{4}$	217–223	4–4.4	[81,82]	18	[81]
	*δ*-HfN	$Fm\bar{3}m$	NaCl	260		[80]	16.3–19.5	[1,80]
	c-Hf${}_{3}$N${}_{4}$${}^{*}$	$I\bar{4}3d$	c-Th${}_{3}$P${}_{4}$	227–241	4–5.3	[83]	DFT: 21.3/18.7	[84]
Group V	VN	$Fm\bar{3}m$	NaCl	265(5)		[85]	6–15	[1,85,86]
	*δ*-NbN	$Fm\bar{3}m$	NaCl	348 GPa		[80]	13.3–20.0	[1,80]
	*β*-Ta${}_{2}$N	$P6_{3}/mmc$	hcp	360(3)		[87]		
	*ϵ*-TaN	$P\bar{6}2m$	CoSn	288(6)	4.7(0.5)	[88]	24.7	[79]
	*η*-Ta${}_{2}$N${}_{3}$${}^{*}$	Pbnm	U${}_{2}$S${}_{3}$	319(6)		[56]	16	[32]
Group VI	CrN	$Fm\bar{3}m$	NaCl	DFT: 340–430		[85]	13–17	[49]
	hp-CrN	Pnma		243(10)		[85]		
	Cr${}_{2}$N	$P6_{3}/mmc$	hcp	275(23)	2.0(2.0)	[88]	15.7	in [77]
	*δ*-MoN	$P6_{3}mc$	FeS (2H)	345(9) GPa	3.5(3)	[88,89]	38.5	[5]
	*γ*-Mo${}_{2}$N	$Fm\bar{3}m$	NaCl	301–304	4	[55,89]	35.7	[77]
	WN	$P\bar{6}m2$	WC				30(5)	[50]
	W${}_{2}$N	$Fm\bar{3}m$	NaCl	240(10)	11.7(1.6)	[74]	31(3)	[50]
Group VII	Mn${}_{3}$N${}_{2}$	I4/mmm	IrU${}_{2}$C${}_{2}$					
	Re${}_{3}$N${}^{*}$	$P\bar{6}m2$		395(7)		[60]		
	Re${}_{2}$N${}^{*}$	$P6_{3}/mmc$	S${}_{2}$Mo	401(10)		[60]		
Group VIII	Fe${}_{2}$N	Pbcn	PbO${}_{2}$					
	*ϵ*-Fe${}_{7}$N${}_{3}$	$P6_{3}/mmc$	hcp	168(10)	5.7(1.5)	[90]		
	$\gamma^{\prime }$-Fe${}_{4}$N	$Fm\bar{3}m$	Fe${}_{4}$N	155(3)		[90]		
	*ϵ*-Fe${}_{3}$N	P312		172(4)	5.7	[91]	7.4(10)	[91]
	OsN${}_{2}$${}^{*}$	Pnnm	o-FeS${}_{2}$	358(6)	4.67	[63]		
	Co${}_{2}$N	Pnnm	CFe${}_{2}$					
	IrN${}_{2}$${}^{*}$	$P2_{1}/c$	CoSb${}_{2}$	428(12)		[63]		
	Ni${}_{3}$N	$P6_{3}22$	O${}_{3}$Re					
	PdN${}_{2}$${}^{*}$	$F\bar{4}3m$	c-FeS${}_{2}$					
	PtN${}_{2}$${}^{*}$	$F\bar{4}3m$	c-FeS${}_{2}$	354–372	4–5.26	[65]		
Group II	Zn${}_{3}$N${}_{2}$	$Ia\bar{3}$	O${}_{3}$Mn${}_{2}$	228(2)		[92]		

## 5. Transition Metal Carbides

The structure-property relations of the carbides of groups IV–VI transition metals have been summarized by Pierson [1]. Carbides of group IV (Ti, Zr, Hf) and group V (V, Nb, Ta) elements have metal-like properties, such as a high thermal and electrical conductivity. They are opaque and have a metallic luster, but they also have ceramic-like properties, such as high melting points, high hardness and large bulk moduli. In the group IV metal carbides, the melting point of the carbide is nearly twice as high as that of the metal, in the group V metal carbides it is about 1.5 times higher, and in the group VI metal carbides the metals actually have a slightly higher melting temperature than the carbides. This combination of properties and their chemical inertness make them interesting candidate materials for applications such as cutting and grinding tools, coatings and diffusion barriers.

Binary carbides are known for all transition metals of periods 4–6 except for Cu, Zn, Tc, Rh, Pd, Ag, Cd, Ir, Au and Hg (Figure 1) [51]. The carbides RuC and OsC have been reported in WC-type structure by Kempter and Nadler [93]. However, these findings are discussed controversially in the literature as DFT modelling results in an instability of the WC structure for these compounds [94,95,96].

The synthesis of transition metal carbides from the elements in the laser-heated DAC is less straightforward as it depends on the mixing of the starting materials. To obtain an homogeneous mixture is complicated by the small size of the sample, and especially in case of heavy transition metals and light carbon by the difference in weight. A further aspect, which might be worth to be studied, is the unknown influence of the state of carbon at different (p,T)-conditions, like graphite or diamond, on the reaction process. In general, only part of the metal and graphite react and the remaining portions of the starting material can be observed throughout the experiment. After the first such synthesis of PtC [97] we have reacted a number of transition metals (Sc, Ti, Ta and Re) with graphite in the laser-heated DAC (Figure 1). Recently, the reaction of metals with carbon diffusing out of the diamond was reported from melting studies [10,11].

### 5.1. Scandium Carbides

**Figure 11:**
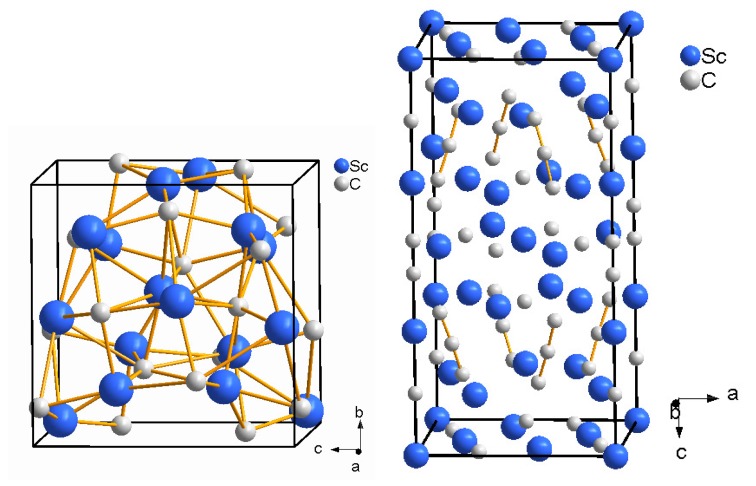
Crystal structures of Sc${}_{4}$C${}_{3}$ ($I\bar{4}3d$, Th${}_{3}$P${}_{4}$-structure type, left [101]) and Sc${}_{3}$C${}_{4}$ (P4/mnc, right [104]).

The Sc-C system contains numerous phases at ambient pressure. A cubic phase with composition between ScC and ScC${}_{2}$ has a NaCl-type structure ($Fm\bar{3}m$) and the lattice parameter varies depending on the composition [98,99,100]. Further cubic phases, which have been proposed, are Sc${}_{2}$C${}_{3}$ ($I\bar{4}3d$, Pu${}_{2}$C${}_{3}$-structure type with *a* = 7.205 Å [100]), Sc${}_{4}$C${}_{3}$ ($I\bar{4}3d$, Th${}_{3}$P${}_{4}$-structure type, *a* = 7.207 Å [101], Figure 11, left) and Sc${}_{13}$C${}_{10}$ (unknown structure type, *a* = 8.53 Å [102]). A tetragonal phase was first reported as Sc${}_{15}$C${}_{19}$ ($P\bar{4}2_{1}c$, *a* = 7.50 Å and *c* = 15.00 Å [103]) but, based on single crystal X-ray diffraction experiments, it was later concluded that the correct composition was Sc${}_{3}$C${}_{4}$ (P4/mnc,*a* = 7.4873 Å and *c* = 15.025 Å, Figure 11, right [104]). Experiments in this system are further complicated by the strong affinity of scandium for oxygen. Even nominally pure Sc metal will contain small amounts of oxygen. It was also reported that stoichiometric ScC with NaCl-type structure cannot form either due to an intrinsic non-stoichiometry or due to the possible formation of Sc${}_{2}$OC [99,105]. The NaCl-type ScC structure is stabilized by an oxygen incorporation and vice versa NaCl-type ScO by carbon incorporation [105,106,107,108]. Hence, mixed crystals of scandium oxycarbides ScC${}_{x}$O${}_{y}$ will form. This phase is in equilibrium with Sc and Sc${}_{2}$O${}_{3}$ in an oxygen-rich environment, while it is in equilibrium with Sc${}_{3}$C${}_{4}$ in a carbon-rich environment [106].

We have investigated the reaction of Sc and C in the laser-heated DAC using scandium foil and flakes as starting materials with the estimated composition Sc${}_{0.87(6)}$O${}_{0.13(6)}$ and excess carbon [33]. Already at the lowest pressure of 9 GPa the reaction occurred immediately with low laser power (about 1000 K) and was completed on further laser heating. Tetragonal Sc${}_{3}$C${}_{4}$ was identified as the main reaction product. Increasing the laser power and temperature (≈1600–2000 K) led to the observation of an additional cubic phase (Sc${}_{2}$C${}_{3}$ or Sc${}_{4}$C${}_{3}$, which only differ by the carbon positions) with *a* = 7.18–7.20 Å at ambient conditions, and of an unknown ScC${}_{x}$ phase, which could be indexed with an orthorhombic unit cell (Pmmm, *a* = 14.7194(7) Å, *b* = 11.9111(7) Å, and *c* = 5.3862(4) Å at 9 GPa). *α*-Sc was still present. Additionally, the formation of cubic ScO${}_{x}$C${}_{y}$ was observed, which is explained by the presence of oxygen in the starting material. A composition between ScO${}_{0.39}$C${}_{0.50}$ and ScO${}_{0.39}$C${}_{0.56}$ was estimated from a comparison of the unit cell parameters at ambient conditions with those published by Karen *et al.* [106]. From DFT-based calculations a higher stability of the Sc${}_{4}$C${}_{3}$ phase was proposed in a scandium-saturated environment with respect to the formation of Sc${}_{2}$C${}_{3}$. All the phases remained stable during heating at 14 GPa and after recovering to ambient conditions.

### 5.2. Titanium Carbide

Cubic *δ*-TiC (B1, $Fm\bar{3}m$, *a* = 4.327(4) Å) is a typical representative of an interstitial transition metal carbide. Similarly to most carbides, the structure can accommodate a large concentration of defects on the “interstitial” carbon positions, leading to substoichiometric TiC${}_{x}$, with 0.5 <x< 1. Lattice parameters and physical properties depend on the defect concentration [1]. Possible ordering schemes of the defects have been discussed in the literature [109]. In a recent *in situ* neutron scattering study at ambient pressure and high-temperature, in which the elements were reacted, only the cubic *δ*-phase was observed [110].

**Figure 12:**
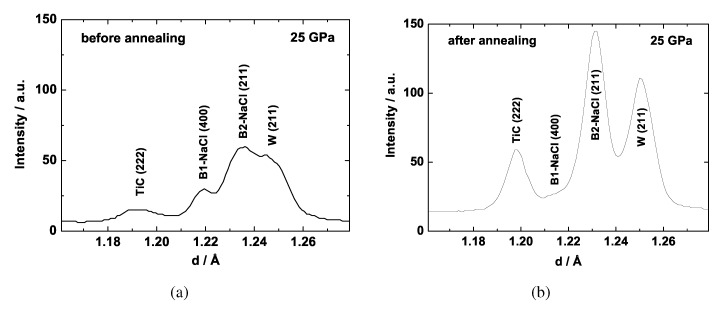
Laser-heating a mixture of Ti and graphite, thermally insulated by NaCl, at 25 GPa leads to a mixture of TiC and NaCl, where the B1 and B2 polymorphs coexist at these conditions [110]. Diffraction lines of tungsten stem from the gasket. Right: Annealing with low laser power yields an improved diffraction pattern with sharp reflections. The amount of the B1 polymorph of NaCl is significantly reduced.

The synthesis of TiC at ambient pressure has been studied with various methods (see summary in Winkler *et al.* [110]) and the physical properties of TiC at ambient pressure have been extensively documented [1]. In an initial high-pressure study of TiC a cubic to rhombohedral phase transition was found to occur at around 18 GPa by Dubrovinskaia *et al.* [111]. This was deduced from the observation of a splitting of the cubic (111) reflection. In a more recent study [112], we have synthesized *δ*-TiC by reaction of the elements at 15 GPa and about 1600–2000 K. At these conditions we observed small amounts of trigonal TiC${}_{x}$ (ICSD #65938 [51]), which however was not detectable at higher pressures and temperatures. In contrast, *δ*-TiC was still present at 25 GPa and about 2000 K. We obtained diffraction patterns at about 25 GPa on heated and annealed samples. After laser heating most of the titanium within the sample had reacted with the graphite to form cubic TiC. No further phases were observed, except for the pressure medium (NaCl) and the gasket (W). Annealing was achieved by scanning the sample from one side with low laser power. This greatly reduced residual strain induced by deviations from hydrostaticity, which is shown for the regions of the cubic (222) reflection of TiC in Figure 12.

The FWHM of the (111) and (222) reflections after annealing were 0.03 Å, and hence no broadening or splitting was detectable. The (111) slightly overlaps with B1-type NaCl-(200), but the non-overlapping (222) also clearly shows no splitting. We further investigated the proposed rhombohedral distortion theoretically using DFT. Even for pressures as high as 40 GPa, the ground state structure remained cubic and a rhombohedral distortion was always unstable. Hence, the results obtained from experiments and model calculations in the more recent study indicate that cubic TiC is the stable phase up to 25 GPa and at least 2000 K and does not undergo a structural phase transition.

The lattice parameter of the recovered TiC sample is *a* = 4.3238(6) Å. Fully stoichiometric TiC has a lattice parameter of 4.327 Å and a carbon deficiency causes the lattice parameter to shrink down to 4.30 Å for TiC${}_{0.5}$ [1]. Hence, the sample synthesized at high-(p,T) conditions seems to be fully stoichiometric.

### 5.3. Iron Carbides

The diffusion of carbon stemming from the diamond anvils of the pressure cell into Fe${}_{1-x}$Ni${}_{x}$ (0.1 <x< 0.22) alloys in the laser-heated DAC was reported already at low temperatures of 1700–1800 K during melting experiments of these alloys [11]. The unexpected occurrence of the fcc-structured phase in addition to the bcc-structured Fe,Ni phase in all experiments between 15–45 GPa and 1450–2600 K indicated the diffusion and incorporation of carbon into the alloy. This probably led to the shift of the *fcc-bcc* phase boundary towards lower temperatures forming carbon-bearing bcc- and fcc-structured (Fe,Ni)C phases.

### 5.4. Tantalum Carbides

**Figure 13:**
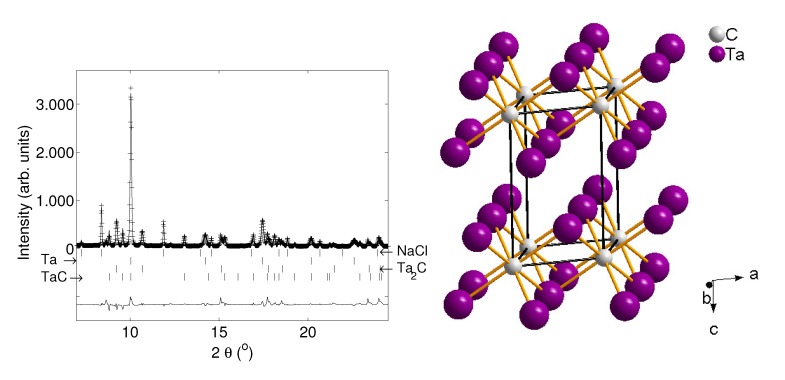
Left: X-ray diffraction pattern (*λ* = 0.4130 Å) of the recovered sample of TaC and Ta${}_{2}$C after laser heating at 11 GPa. Right: Crystal structure of Ta${}_{2}$C ($P\bar{3}m1$, CdI${}_{2}$-type structure [51]).

In the Ta-C system four phases are stable at ambient pressure up to 2423 K: Ta, TaC${}_{1-x}$, Ta${}_{2}$C${}_{1-x}$ and Ta${}_{4}$C${}_{3-x}$ [113]. In a recent experiment we laser heated a mixture of the elements at pressures between 8.6 and 14.3 GPa and temperatures up to 2300 K. It always resulted in the formation of cubic TaC ($Fm\bar{3}m$, NaCl-type, *a* = 4.4353(5) Å), while Ta${}_{2}$C ($P\bar{3}m1$, CdI${}_{2}$-type, *a* = 3.0969(3) Å, *c* = 4.9388(5) Å, Figure 13) was formed concomitantly in some experiments only (Figure 13). Ta and graphite begin to react around 1100 K at ambient pressure conditions and the reaction temperature increases with increasing pressure.

The reaction of tantalum with carbon diffusing out from the surface of the diamond anvils of the pressure cell was observed during melting studies of tantalum by Dewaele *et al.* [10]. These experiments show the high stability of cubic TaC at extreme pressures and temperatures of up to 90 GPa and 3600 K. The carbidation of metals by carbon diffusion from the diamonds has a strong influence on the measured melting curves and leads to an underestimation of the melting curves of the metals. This may be the reason for the large discrepancies of various published melting curves of metals from laser-heated DAC experiments.

**Figure 14:**
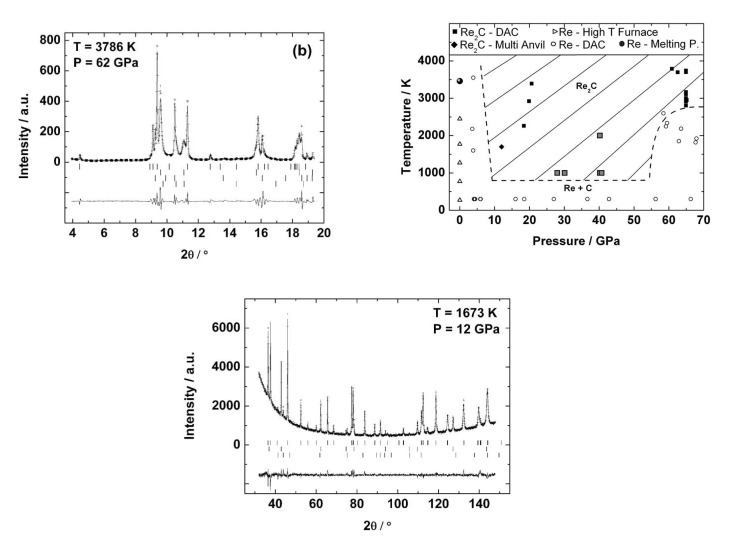
Top left: Powder X-ray diffraction pattern of the reacted Re${}_{2}$C sample in a DAC at high (p,T) (*λ* = 0.3738 Å). Top right: (P,T)-field in which Re${}_{2}$C can be synthesized. Bottom: Powder X-ray diffraction pattern and Rietveld refinement of a sample synthesized in a multi anvil press (*λ* = 1.5406 Å).

### 5.5. Rhenium Carbide

**Figure 15:**
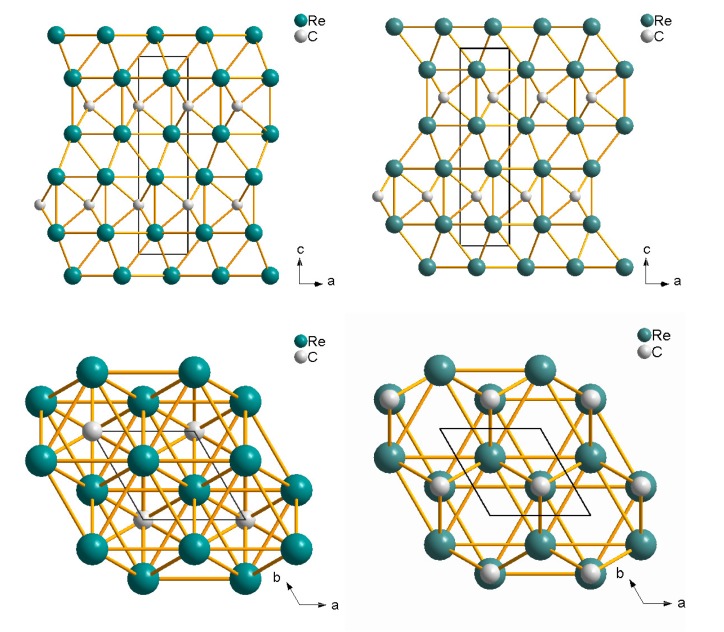
Crystal structure of Re${}_{2}$C ($P6_{3}/mmc$) of anti-MoS${}_{2}$-type after Juarez-Arellano *et al.* [62] (left) and of anti-ReB${}_{2}$-type after Zhao *et al.* [115] (right). Note the different carbon positions.

In two recent studies [62,114] we have shown that a novel rhenium carbide can be synthesized from the elements at high-(p,T) conditions. No reaction of the elements was observed at a lower pressure of around 4 GPa on laser heating. However, the rhenium carbide phase was formed at pressures between 18 and 67 GPa and temperatures between about 1000 and 3800 K. Indexing the powder X-ray diffraction patterns was not straightforward as two argon polymorphs coexisted at the synthesis conditions (Figure 14, top left), but we were able to outline the (p,T) space in which the new phase was stable (Figure 14, top right). Multi-anvil cell experiments yielded enough sample to locate the rhenium atoms through a Rietveld refinement (Figure 14, bottom), while it was impossible to locate the light carbon atoms. Hence, DFT-based calculations were used to calculate the relative stabilities of compounds in which the available interstices were filled with carbon atoms. This allowed us to conclude that the new rhenium carbide had a composition Re${}_{2}$C ($P6_{3}/mmc$, *a* = 2.8425(1) Å, *c* = 9.8584(1) Å, Figure 15, left) and is isostructural to the anti-MoS${}_{2}$ structure, and hence also to Re${}_{2}$N (see Figure 8).

In order to investigate the stability of Re${}_{2}$C, Zhao *et al.* [115] have performed synthesis experiments at moderate pressures of 1–6 GPa and temperatures 873–1873 K *via* reaction sintering using a large-volume press. Different Re/C ratios resulted only in one kind of Re-C compound, *i.e*., the above reported Re${}_{2}$C, however with a measured composition of Re${}_{2}$C${}_{0.924}$. Another structural model, the anti-ReB${}_{2}$-type one with carbon occupying a different site compared to the structure proposed by Juarez-Arellano *et al.* [62], was proposed on the basis of theoretical calculations (Figure 15, right). The authors found this phase stable already at pressures of 2 GPa (at 1673–1873 K) to 6 GPa (at 1073–1873 K). This (p,T)-range was not studied by [62,114] except for one (p,T)-point at about 4 GPa and 1600 K, where Juarez-Arellano *et al.* [62] did not observe the formation of Re${}_{2}$C, while Zhao *et al.* [115] observed a mixture of Re${}_{2}$C with Re and carbon.

### 5.6. Platinum Carbide

The first experiment to synthesize a transition metal carbide by reaction of the elements in the laser-heated DAC was the formation of platinum carbide [97]. A mixture of platinum and carbon powder was ground together and loaded with argon as pressure-transmitting medium and thermal insulation. The platinum carbide phase was formed at pressures between 60 and 121 GPa at temperatures of about 2600 K. However, on heating at a lower pressure of 47 GPa it decomposed, which points to the crucial role of pressure for the synthesis. The chemical composition is likely to be PtC with NaCl-type structure ($Fm\bar{3}m$, *a* = 4.5798 Å at 85 GPa and 2600 K) [97].

### 5.7. Mechanical Properties of Transition Metal Carbides

The experimentally obtained values for the bulk modulus and hardness of several binary transition metal carbides are summarized in Table 2. The bulk moduli of the scandium carbides are comparatively low, and range from 100 to 200 GPa, in accordance with the more ionic, salt-like character of the group III carbides (see Pierson [1]). The increasing number of *d* electrons within a period is correlated with a decrease of the compressibility from the group IV to the group VI carbides. The same is observed for the carbides within a group. Hence, the heavier transition metals tend to form less compressible carbides. The lowest experimentally determined compressibilities ($B_{0}$ > 400 GPa) are observed for WC and Re${}_{2}$C. PtC has $B_{0}$ = 339 GPa [97]. In order to complete our understanding of the structure-property relations of the period 6 transition metal carbides, it is now of great interest to experimentally determine the bulk modulus of osmium carbide where no unambiguous structural data exist. The high-temperature synthesis of OsC in the WC-structure type was claimed by Kempter and Nadler [93], but only a set of *d*-spacings was reported. Numerous theoretical studies show that this OsC would be unstable, but they partially contradict each other and different structure types have been predicted for the high-(p,T) polymorph (e.g., [94,95,114]).

The interstitial and intermediate carbides of groups IV–VII [1], which are summarized in Table 2, all have hardness between about 17 and 35 GPa. As has been noted above, these values strongly depend on the measurement technique and sample microstructure, and hence no clear trends in structure-composition-hardness relations are currently obvious (see discussion below).

**Table 2:** Properties of selected transition metal carbides. $B_{0}$ is the bulk modulus and $B^{\prime }$ its pressure derivative. ${}^{*}$ indicate phases obtained at high-(p,T) only.

	Compound	Space group	Structure	$B_{0}$ (GPa)	$B^{\prime }$	Ref.	Hardness (GPa)	Ref.
Group III	ScO${}_{x}$C${}_{y}$	$Fm\bar{3}m$	NaCl	190(90)		[33]		
	Sc${}_{4}$C${}_{3}$	$I\bar{4}3d$	Th${}_{3}$P${}_{4}$	157(2)		[33]		
	Sc${}_{3}$C${}_{4}$	P4/mnc		144(3)		[33]		
	ScC${}_{x}$${}^{*}$	Pmmm		105(1)		[33]		
Group IV	TiC	$Fm\bar{3}m$	NaCl	233–268	4–6.5	[111,116,117,118,119,120,121,122]	28–35	[1]
	ZrC	$Fm\bar{3}m$	NaCl	207-223		[1,116]	26(2)	[1,77]
	HfC	$Fm\bar{3}m$	NaCl	242		[123]	26.1	[1]
Group V	VC	$Fm\bar{3}m$	NaCl	258(11), 390	4.5(6)	[1,124]	27–29	[77,125]
	NbC	$Fm\bar{3}m$	NaCl	266.7–340		[1,123,126,127,128]	19.6–24	[1,77]
	TaC	$Fm\bar{3}m$	NaCl	317–345		[120,123,129,130,131]	13.5–18	[1,77,113]
	TaC${}_{0.90}$	$Fm\bar{3}m$	NaCl	217		[132]		
Group VI	Cr${}_{7}$C${}_{3}$	Pnma	Cr${}_{7}$C${}_{3}$	DFT: 300–315	4.26	in [133]	16	[134]
	Cr${}_{23}$C${}_{6}$	$Fm\bar{3}m$	Cr${}_{23}$C${}_{6}$	DFT: 282–300		in [133]	14.5	[134]
	Cr${}_{3}$C${}_{2}$	Pnma	Cr${}_{3}$C${}_{2}$	DFT: 313–333		in [133]	10–18	[1]
	Mo${}_{2}$C	Pbcn	Fe${}_{2}$N	307(5)	6.2(3)	[135]	14–24.5	[1,135,136]
	WC	$P\bar{6}m2$	WC	329-439	4-4.7	[1,123,137,138]	22–28	[1,29,139]
Group VII	Re${}_{2}$C${}^{*}$	$P6_{3}/mmc$	S${}_{2}$Mo	405(30)	4.6	[114]	17.5	[115]
Group VIII	Fe${}_{3}$C	Pnma	Fe${}_{3}$C	174–175	4.8–5.2	[140,141]	8–11	[134]
	Fe${}_{7}$C${}_{3}$	$P6_{3}mc$	Cr${}_{7}$C${}_{3}$	253	3.6	in [142]		
	PtC${}^{*}$	$Fm\bar{3}m$	NaCl	301-339	4–5.2	[97]		

## 6. Transition Metal Borides

Similar to the respective mononitrides, the monoborides have melting points about 1.5 times higher than the metal for group IV and slightly higher than the metal for group V borides. In contrast to the group VI nitrides, however, the monoborides of group VI can still be termed refractory with melting points above 2200 K which are close to those of the pure metals.

The synthesis of transition metal borides at ambient pressure is relatively straightforward by heating a mixture of the elements above ≈1500 K. Hence, binary borides have been reported for all transition metals from periods 4–6 with the exception of Cu, Zn, Tc, Cd and Hg (Figure 1) [51]. Cu and Zn have only been incorporated as minor amounts in rhombohedral *β*-boron, e.g., CuB${}_{23}$, CuB${}_{28}$, ZnB${}_{25}$ [51]. The existence of Ag and Au borides is discussed controversially. Several synthesis attempts of AgB${}_{2}$ have failed [143,144] after the synthesis had been reported by Obrowski [145]. The interest in AgB${}_{2}$ is due to the prediction that it would have a very high transition temperature of 59 K into the superconducting state [146].

Only one synthesis experiment has been published in a laser-heated DAC, namely the synthesis of TaB${}_{2}$ by our group [147]. Two further synthesis experiments in the Ti-B and Re-B systems have been successful and will be discussed here. Other borides such as Nb${}_{1-x}$B${}_{2}$, Ta${}_{1-x}$B${}_{2}$, and Mo${}_{1-x}$B${}_{2}$ have been synthesized at high-(p,T) conditions in a multi-anvil cell at 5 GPa and 1473 K in order to study their superconductivity as a function of composition [148]. It is obvious that these syntheses could also be performed in a laser-heated DAC.

### 6.1. Titanium Borides

TiB${}_{2}$ has been synthesized by several groups at high-(p,T) conditions. Miyamoto *et al.* [149] obtained dense TiB${}_{2}$ by high-pressure self-combustion sintering processing after the electric ignition at one end of a powdered mixture of Ti and B at 3 GPa. Bhaumik *et al.* [150] prepared TiB${}_{2}$ by high-pressure sintering (HPS) of premixed powders and by high-pressure self-combustion synthesis (HPCS) from the elemental constituents. The sintering and synthesis experiments were carried out at 3 GPa and 2250–2750 K. It was reported that a high sintering temperature of 2750 K was required to obtain 98% dense TiB${}_{2}$ samples by HPS and a minimum ignition temperature of 2250 K was required in HPCS to make the reaction self-sustaining. Due to the exothermicity of the reaction of the mixed elements, the reaction is triggered at the ignition temperature at any part of a compact sample, like in this example by passing current through a graphite heater for 5 s at the outer rim of the sample, and then self-propagates throughout the sample.

**Figure 16:**
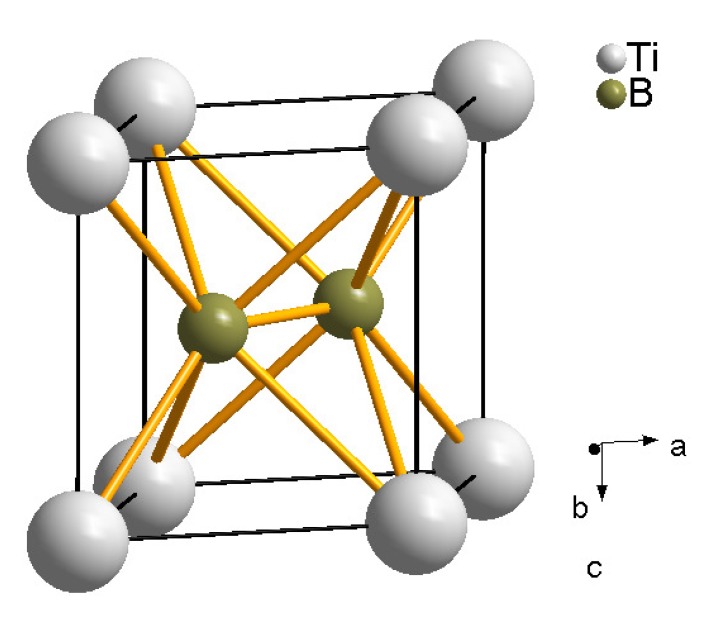
Crystal structure of TiB${}_{2}$ of AlB${}_{2}$ structure type.

**Figure 17:**
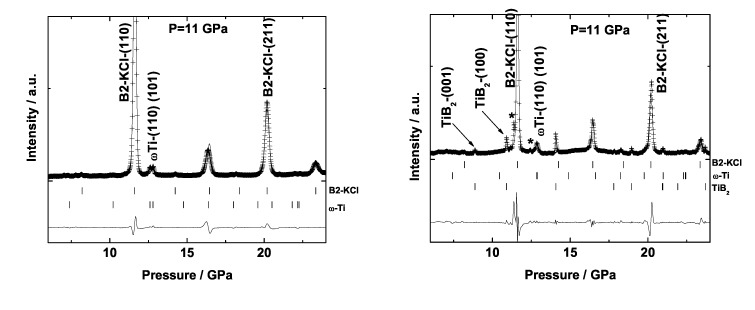
X-ray diffraction pattern before laser heating (left) and after laser heating (right) a mixture of titanium and boron in a DAC (*λ* = 0.4958 Å).

In a recent experiment we synthesized TiB${}_{2}$ (AlB${}_{2}$-type, P6/mmm, *a* = 3.03 Å and *c* = 3.238 Å, Figure 16) in a laser-heated DAC from the elements using titanium foil and boron powder. We observed that at pressures between 10 and 11 GPa and very weak laser heating (≈1000 K) *ω*-titanium and boron reacted immediately (Figure 17). After further laser heating the following phases could be observed: TiB${}_{2}$, *ω*-titanium and KCl in its high-pressure CsCl-type structure (pressure medium) are the main phases, while most of the additional reflections could be indexed by Ti${}_{2}$B${}_{5}$ (ICDD 06-0528, $P6_{3}/mmc$,*a* = 2.9331(5) Å and *c* = 13.910(5) Å [151]). However, as it can be seen in Figure 17, the low intensity of the Bragg reflections of *ω*-titanium (before and after laser heating) compared to the KCl peaks is due to the very small amount of sample in the X-ray beam with respect to the KCl pressure medium. This makes it difficult to follow the reaction at high-(p,T) conditions and to confidently identify the products of the reaction. Therefore further experiments have to be performed.

### 6.2. Tantalum Boride

**Figure 18:**
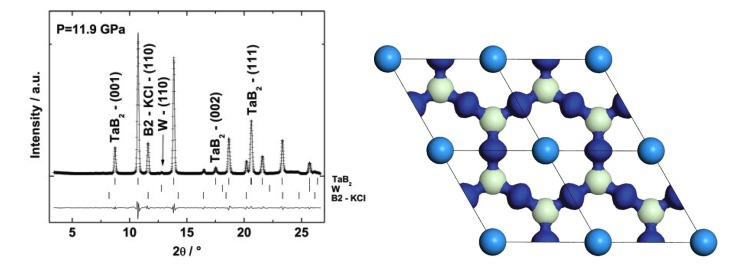
Left: X-ray diffraction pattern after laser heating (*λ* = 0.4958 Å). Right: Electron density difference isosurface at 0.07 $e^{-}$/Å${}^{3}$. The medium grey (medium blue) spheres at the corners of the unit cells are Ta atoms. The light grey (light green) spheres represent B-atoms at *z* = $\frac{1}{2}$. The dark grey (dark blue) isosurface shows the difference between the self-consistent charge distribution and the electron density of superposed, non-interacting atomic electron densities. The large charge accumulation between the boron atoms is indicative of the formation of covalent bonding. The Ta-B bonds also have a significant covalent character, the corresponding bond population is 0.75 $e^{-}$/Å${}^{3}$ [147].

Several tantalum borides of different stoichiometries have been reported at ambient pressure and were summarized in our recent study [147], namely in increasing order of boron content Ta${}_{2}$B, Ta${}_{3}$B${}_{2}$, TaB, Ta${}_{5}$B${}_{6}$, Ta${}_{3}$B${}_{4}$, TaB${}_{2}$. We have shown that on laser heating a sample of tantalum foil and boron powder at pressures of 12–24 GPa and temperatures of 1600–2000 K tantalum and boron reacted immediately, already after a brief irradiation with a weak laser beam, and that pure tantalum was not present any more in the sample. The same synthesis product, TaB${}_{2}$ (AlB${}_{2}$-type, P6/mmm, *a* = 3.1018(2) Å, *c* = 3.2836(4) Å) as a single stable phase, was observed independently of the pressure (11.9 or 23.7 GPa) or the temperature used (laser heating with different laser power was attempted) (Figure 18, left) [147].

From our (p,V)-data we observed that the *c*-axis is substantially more compressible than the *a*-axis. This behavior was expected due to the graphite-like boron layers which are characteristic of the TaB${}_{2}$ structure. The TaB${}_{2}$ structure has strong covalent bonds between the boron atoms. A population analysis gives a very high value of 2.25 $e^{-}$/Å${}^{3}$ for the B-B bonds. This can be visualized by plotting electron density difference isosurfaces (Figure 18, right). Compression to 30 GPa does not significantly change the bond populations.

### 6.3. Rhenium Borides

Without doubts, rhenium diboride, ReB${}_{2}$ ($P6_{3}/mmc$, *a* = 2.9 Å and *c* = 7.478 Å), is one of the most studied transition metal borides due to its hardness and incompressibility. Since the synthesis of pure ReB${}_{2}$ was reported [152], several synthesis methods have been employed to obtain ReB${}_{2}$ as powder, single crystals or thin films (e.g., solid state reaction, self-propagating high-temperature synthesis, arc melting, zone melting, optical floating zone furnace, pulsed laser deposition [3,152,153,154,155]). However, except for the ReB${}_{2}$ phase the rest of the Re-B system has barely been explored and only few studies have been reported. Kawano *et al.* [156] reported the superconductivity of Re${}_{7}$B${}_{3}$ (T${}_{c}$ = 3.3 K) and Re${}_{3}$B (T${}_{c}$ = 4.8 K), while Takagiwa *et al.* [157] reported the magnetic properties of Re${}_{3}$B.

**Figure 19:**
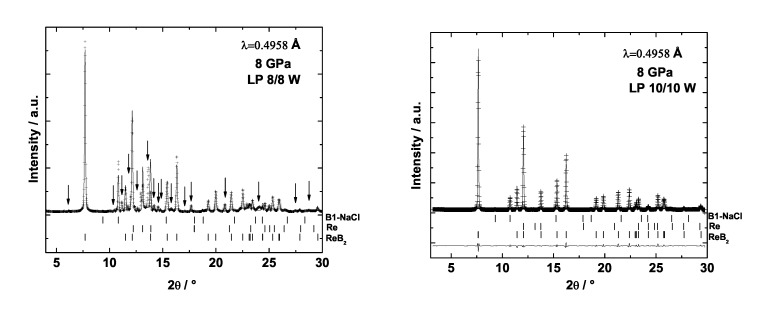
X-ray diffraction patterns after laser heating with different laser power a mixture of rhenium and boron in a DAC (*λ* = 0.4958 Å). Left: Arrows indicate unidentified reflections.

**Figure 20:**
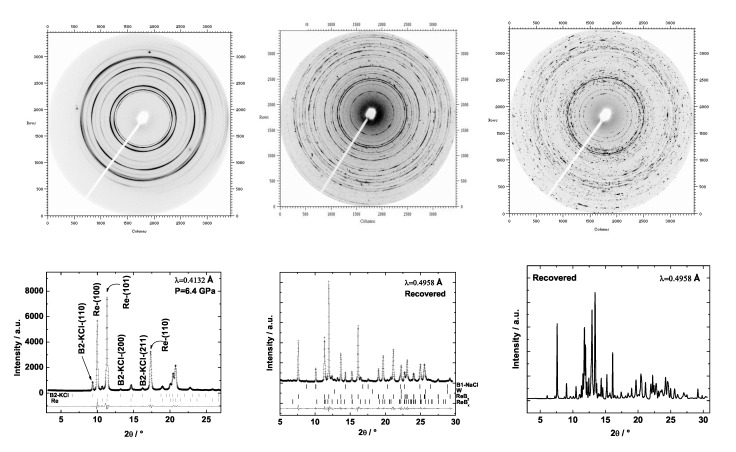
Selected images recorded with a MAR345 image plate detector at the ALS (top row) and corresponding integrated powder X-ray diffraction patterns (bottom row) show the sample before laser heating (left) and from two different recovered samples.

We reacted rhenium foil and boron powder in a laser-heated DAC. We observed that the Re-B system is very complex at high-(p,T) conditions. For example, at 8 GPa and about 1500 K rhenium and boron reacted forming ReB${}_{2}$ but several reflections could not be indexed with the parameters of any of the reported rhenium boride phases (Figure 19, left). However, if the temperature is increased, the unknown reflections disappear and only rhenium and ReB${}_{2}$ coexist. This indicates that the one or more unidentified phases formed at 8 GPa and lower temperature are metastable and that the stable phase is ReB${}_{2}$. Nevertheless, ReB${}_{2}$ is not the only stable phase at high-(p,T) conditions. Selected images recorded with a MAR345 image plate detector and the corresponding integrated powder X-ray diffraction patterns from two recovered gaskets after releasing the pressure from around 21 GPa are shown in Figure 20. The integrated powder diffraction patterns look very different and do not fully coincide either in the position and intensity or in the number of reflections, although the pressure prior to release was nearly the same. This implies that a different number of phases or even various phases were obtained, which might depend on the individual (p,T)-paths of the experiments resulting in distinct equilibriums of phases. Further experiments of this complex system at high-(p,T) conditions promise new and interesting findings.

### 6.4. Mechanical Properties of Transition Metal Borides

The experimentally obtained values for the bulk modulus and hardness of several binary transition metal borides are summarized in Table 3. A comprehensive review about the properties of borides obtained from experiment and theoretical calculations was given by Ivanovskii [158]. All the transition metal borides that have been studied with the exception of Fe${}_{2}$B are very incompressible. The highest bulk moduli (>400 GPa) are reported for the osmium borides. OsB serves as an example where the correlation between high incompressibility and high hardness is not valid, as the hardness is only about 14 GPa [121] (Table 3, Figure 24). However, most transition-metal borides known are hard materials.

## 7. Conclusions

### 7.1. Crystal Chemistry

There are close structural relationships between the group IV–VI carbides and the corresponding nitrides. Most transition metal carbides and nitrides of groups III–VI with a metal/N or metal/C ratio of about 1:1 form compounds with NaCl-type structure (Figure 2), except for WN and WC, which both crystallize in WC-type structure. However, there is a structural diversity for phases with other metal/N or metal/C ratios. A close relationship has also been established for the rhenium compounds, as Re${}_{2}$C, Re${}_{2}$N and Re${}_{3}$N are structurally related and in fact are all equally incompressible. For the Os, Ir, Pt and Pd nitrides and carbides, a structural relationship is not expected due to the occurrence of dinitrogen units in the crystal structure of the respective nitrides (Figure 10). For example, the structures of PtN${}_{2}$ (pyrite-type) and PtC (NaCl-type) are unrelated.

**Table 3:** Properties of selected transition metal borides. $B_{0}$ is the bulk modulus and $B^{\prime }$ its pressure derivative.

	Compound	Space group	Structure	$B_{0}$ (GPa)	$B^{\prime }$	Ref.	Hardness (GPa)	Ref.
Group IV	TiB${}_{2}$	P6/mmm	AlB${}_{2}$	240, DFT: 292	DFT: 3.34	[6,159]	15–45	[1,29]
	ZrB${}_{2}$	P6/mmm	AlB${}_{2}$	317	4	[159]	22.5–35	[1,29]
	HfB${}_{2}$	P6/mmm	AlB${}_{2}$				29.0	[1]
Group V	VB${}_{2}$	P6/mmm	AlB${}_{2}$	322	4	[159]	20.9	[1]
	NbB${}_{2}$	P6/mmm	AlB${}_{2}$				20.9	[1]
	TaB${}_{2}$	P6/mmm	AlB${}_{2}$	336.3(5)	4	[147]	22.6–25.6	[1,160]
Group VI	CrB${}_{2}$	P6/mmm	AlB${}_{2}$				20.5	[1]
	Mo${}_{2}$B${}_{5}$	$R\bar{3}m$					23.0	[1]
	MoB${}_{3}$	$P6_{3}/mmc$					31.8	[115]
	W${}_{2}$B${}_{5}$	$R\bar{3}m$	Mo${}_{2}$B${}_{5}$				26.1	[1]
	WB${}_{2}$	$P6_{3}/mmc$		341–372	4–6.4	[2]	38.4(14)/27.7(6)	[2]
	WB${}_{4}$	$P6_{3}/mmc$		304–325		[2,161]	46.2(12)/31.8(12)	[2]
Group VII	ReB${}_{2}$	$P6_{3}/mmc$	ReB${}_{2}$	360–382		[3,162,163]	30–48	[2,3,4,29,162]
Group VIII	bct-Fe${}_{2}$B	I4/mcm	Al${}_{2}$Cu	164(14)	4.4(5)	[164]	14	[134]
	RuB	$P\bar{6}m2$	WC	261–275	4–5.2	[2]	13.6	[158]
	RuB${}_{2}$	Pmmn	RuB${}_{2}$	242–303	4–9.7	[2,162]	19(2)	[162,163]
	OsB	$P\bar{6}m2$	WC	431–453	4–5.8	[2]	14.4(1.1)/10.6(1.3)	[2]
	Os${}_{2}$B${}_{3}$	$P6_{3}/mmc$	Ru${}_{2}$B${}_{3}$	396–443	4–7.1	[2]	21.8(1.5)/14.7(8)	[2]
	OsB${}_{2}$	Pmmn	RuB${}_{2}$	342–395, 297(25)	4–4.4	[2,158,162,163,165,166]	17–37	[2,158,162,163]
	IrB${}_{2}$	P6/mmm	AlB${}_{2}$	DFT: 324	4.3	[167]		
	IrB	$P6_{3}/mmc$		DFT: 346	4.58	[167]		

The crystal structures of most borides are not obviously related to those of the respective carbides and nitrides. For many transition metal borides with a metal/boron ratio of 1:2, especially those of groups IV and V, the AlB${}_{2}$ structure type is predominant (Figure 16). If the metal/boron ratio is 2:1 the Al${}_{2}$Cu-type structure is often formed for the borides.

### 7.2. Non-Stoichiometry

There is a significant influence of the stoichiometry on the compressibility of transition metal carbides, nitrides and borides. This is expected as the lattice parameters, and hence the unit cell volume, also depend strongly on the composition, e.g., TaN${}_{x}$ [168,169] and TaC${}_{x}$ [113]. As an example, a bulk modulus of 217 GPa was reported for TaC${}_{0.9}$ [132], while bulk moduli between 317 and 345 GPa were reported for nearly stoichiometric TaC${}_{x}$ with 0.97 <x< 0.994 (Table 2). The stoichiometry is also related to the hardness of a compound. While positive correlations were observed for, e.g., the group IV carbides TiC${}_{1-x}$, ZrC${}_{1-x}$ and HfC${}_{1-x}$, the group V carbides reach a maximum hardness below stoichiometry [1]. Further examples for the dependence of the hardness on the stoichiometry of nitrides were demonstrated on thin films by Hones *et al.* [50] and Grant *et al.* [49].

From a comparison of the lattice parameters of the transition metal carbides, nitrides, and borides, which were synthesized from the elements in the laser-heated DAC, with literature data and DFT-based computations, we conclude that extreme conditions favor the synthesis of stoichiometric samples, e.g., TiC [112]. However, further studies on the chemical compositions of the reaction products of laser-heated DAC experiments are required to establish this firmly.

### 7.3. Compressibility

The period 6 transition metal carbides, nitrides and borides are predominantly ultra-incompressible. A few of these materials approach and even exceed the bulk modulus of diamond ($B_{0}$ = 442–446 GPa [170,171,172]), e.g., OsB ($B_{0}$ = 453 GPa [2]), Os${}_{2}$B${}_{3}$ ($B_{0}$ = 443 GPa [2]), WC ($B_{0}$ = 329–439 GPa [1,123,137,138]) and IrN${}_{2}$ ($B_{0}$ = 428 GPa [63]) (Table 1, Table 2 and Table 3).

**Figure 21:**
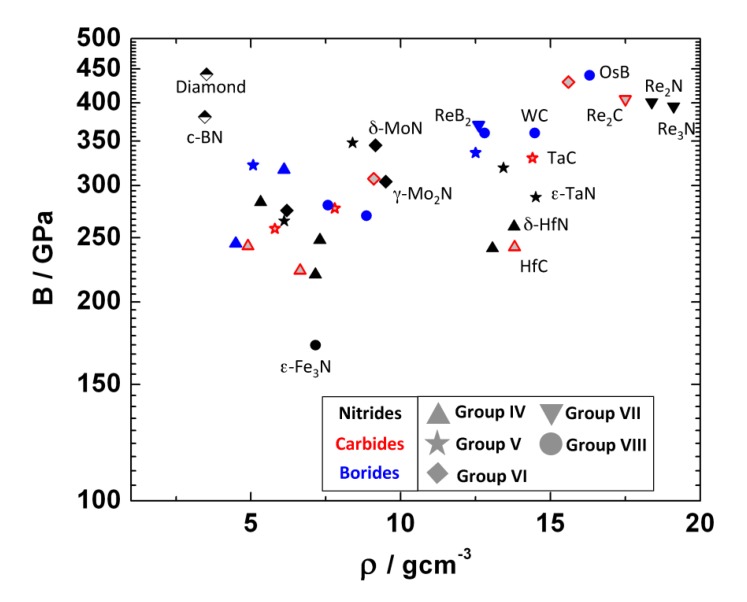
Plot of the bulk moduli of binary transition metal carbides, nitrides and borides versus density in comparison with cubic BN and diamond.

A plot of the bulk modulus versus density of binary transition metal carbides, nitrides and borides shows a slight positive correlation (Figure 21). A correlation between the bulk modulus and density within a structure type was already proposed by Birch’s law [173,174]. This further confirms the approach of materials design, where heavy transition metals are combined with light elements to form ultra-incompressible compounds.

**Figure 22:**
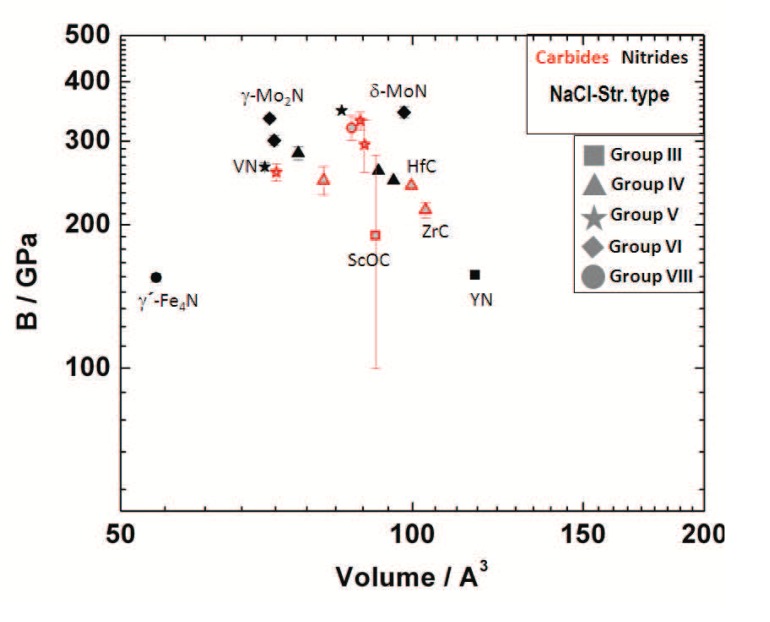
Plot of the unit cell volume of NaCl-type binary transition metal carbides and nitrides versus bulk modulus.

In Figure 22 the unit cell volumes of NaCl-type carbides and nitrides are plotted versus their bulk moduli. The general trend follows an inverse relation. This is expected for a structure type according to Anderson and Nafe [175], who adapted Birch’s law [173,174] to bulk modulus–volume relationships. Only $\gamma^{\prime }$-Fe${}_{4}$N seems to be an outlier, which might be due to the deficiency of nitrogen in the crystal structure.

### 7.4. Hardness

**Figure 23:**
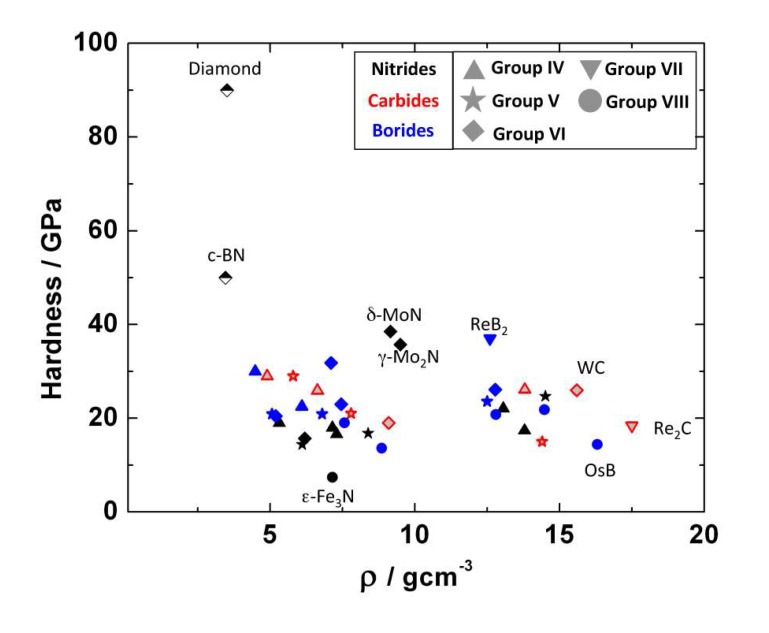
Plot of the hardness versus density of binary transition metal carbides, nitrides and borides in comparison with cubic BN and diamond.

Despite the large effort that was taken on the synthesis and characterization of binary transition metal carbides, nitrides and borides, none of the materials approximates the hardness of diamond (Figure 23), the hardest material known (H${}_{v}$ = 56–102 GPa [176,177]) nor that of the second-hardest material, *c*-BN (H${}_{v}$ = 40–83 GPa [29,178]. There is no obvious correlation between the hardness of a compound and its density (Figure 23). Further, the hardest materials, *i.e*., diamond and *c*-BN do not follow the positive correlation of bulk modulus versus density as they are ultra-incompressible but of least density in Figure 21. They obey another approach of materials design, where the strong covalent bonding between the light atoms accounts for the stiffness of the material.

**Figure 24:**
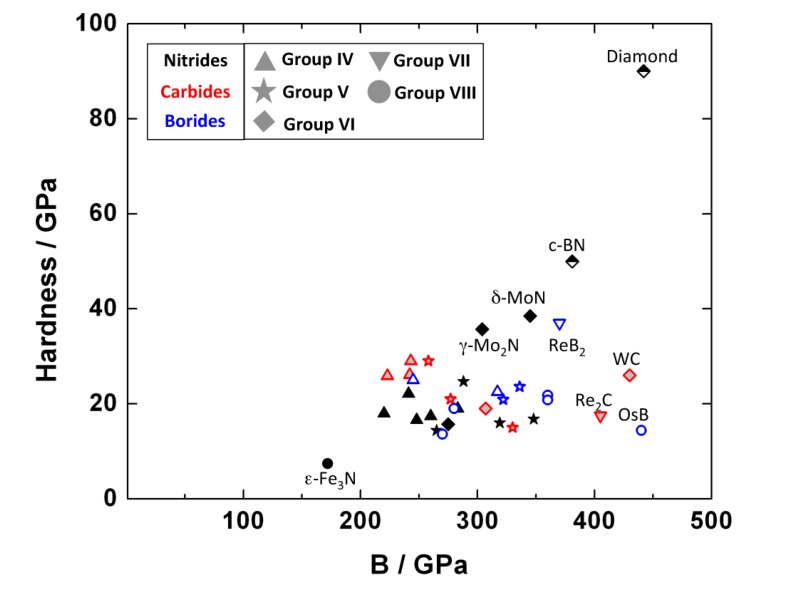
Plot of the hardness versus bulk modulus of various binary transition metal carbides, nitrides and borides in comparison with cubic BN and diamond.

From the plot of the hardness versus the bulk modulus a tendency of positive correlation is visible (Figure 24). However, strong scatter is observed for the compounds reviewed here. Especially WC, Re${}_{2}$C and OsB, which are extremely incompressible with a bulk modulus close to that of diamond, do not show the high hardness which would be expected from a direct correlation with that of diamond. The second-hardest material, cubic *c*-BN ($B_{0}$ = 369 GPa [179]), is even more compressible than the rhenium nitrides, rhenium carbide and rhenium boride (ReB${}_{2}$), OsB${}_{2}$ and PtN${}_{2}$. Further, it is already known from the data of the pure transition metals, which are extremely incompressible but soft, e.g., Os with *B* = 462 GPa and H${}_{v}$ = 4 GPa [180,181], that the incompressibility of a compound is not a direct measure of its hardness. As has been already mentioned above, another point to consider is that the hardness values strongly depend on the measurement technique and sample microstructure [29,31], which may also account for scatter of the respective data.

In summary, the on-going effort to explore the high-(p,T) synthesis and phase stability of binary transition metal carbides, nitrides and borides has led to interesting new results. However, there are obvious gaps in our knowledge and numerous remaining challenges, and undoubtedly further experiments will continue to yield surprising and exciting results.
